# Supplementary material for: Altering translation allows E. coli to overcome chemically stabilized G-quadruplexes
Source: bioRxiv. 2024 Aug 12:2024.08.12.607615. Preprint. [Version 1] doi: 10.1101/2024.08.12.607615 (PMC11343134; doi:10.1101/2024.08.12.607615)
Supplement: Supplement 1 [file media-1.pdf]

SI Figures and Tables for Altering translation allows *E. coli* to overcome chemically stabilized G-quadruplexes

Figures and Tables with legends

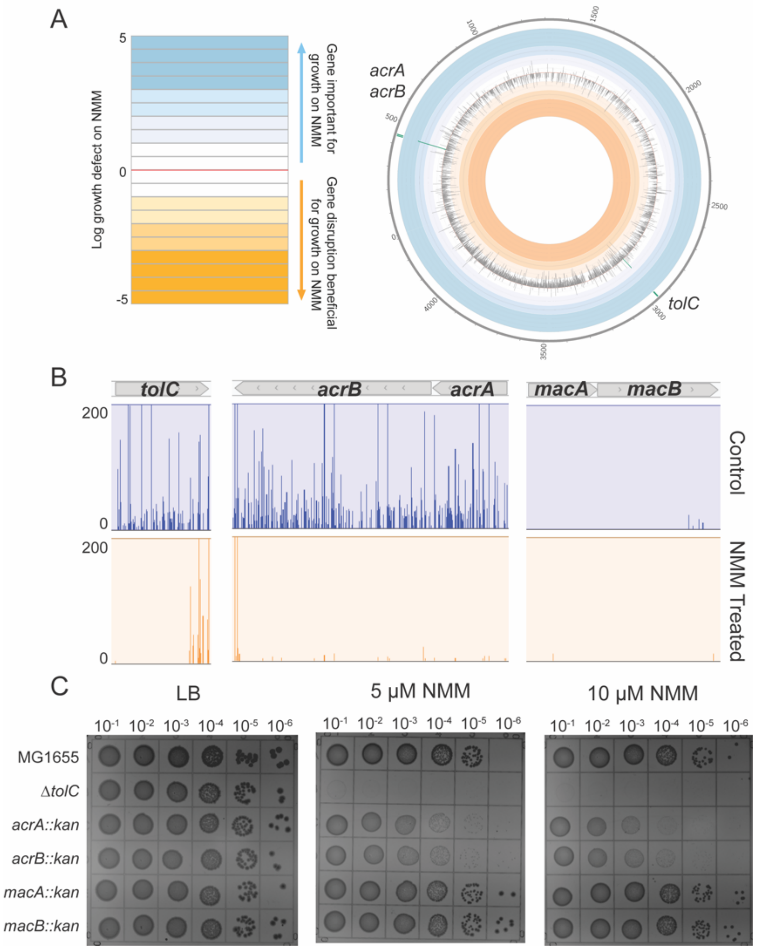

Figure S1: Tn-Seq reveals that *E. coli* are sensitized to NMM when efflux pumps are compromised. (A). Circos plot showing $\log_{10}$(control weighted reads/NMM weighted reads) with genes involved in AcrAB-TolC efflux pump shown in green. **(B).** Mochiview plots of insertions across genes *acrA, acrB, tolC, macA,* and *macB*. **(C)**. Spot dilution plates of LB-agar and increasing concentrations of NMM with strains containing deletions of *tolC*, *acrA, acrB, macA,* and *macB*.

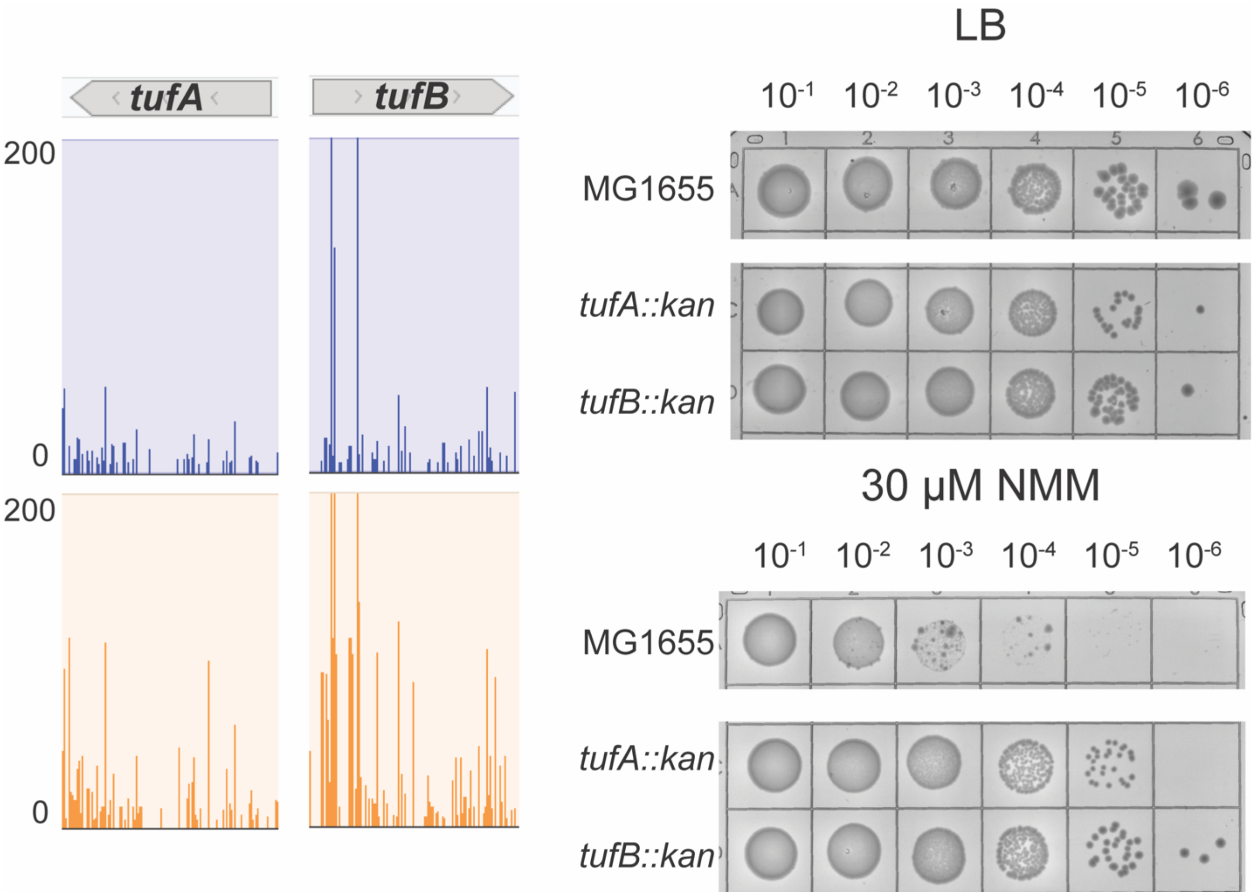

Figure S2: Slowing down translation elongation allows cells to better in the presence of G4 stabilizer. Mochiview insertion plots of *tufA* and *tufB* in the original Tn-seq screen and spot dilution plates containing the *tufA* and *tufB* gene deletions in the MG1655 cell background on LB-agar alone or LB-agar supplemented with NMM.

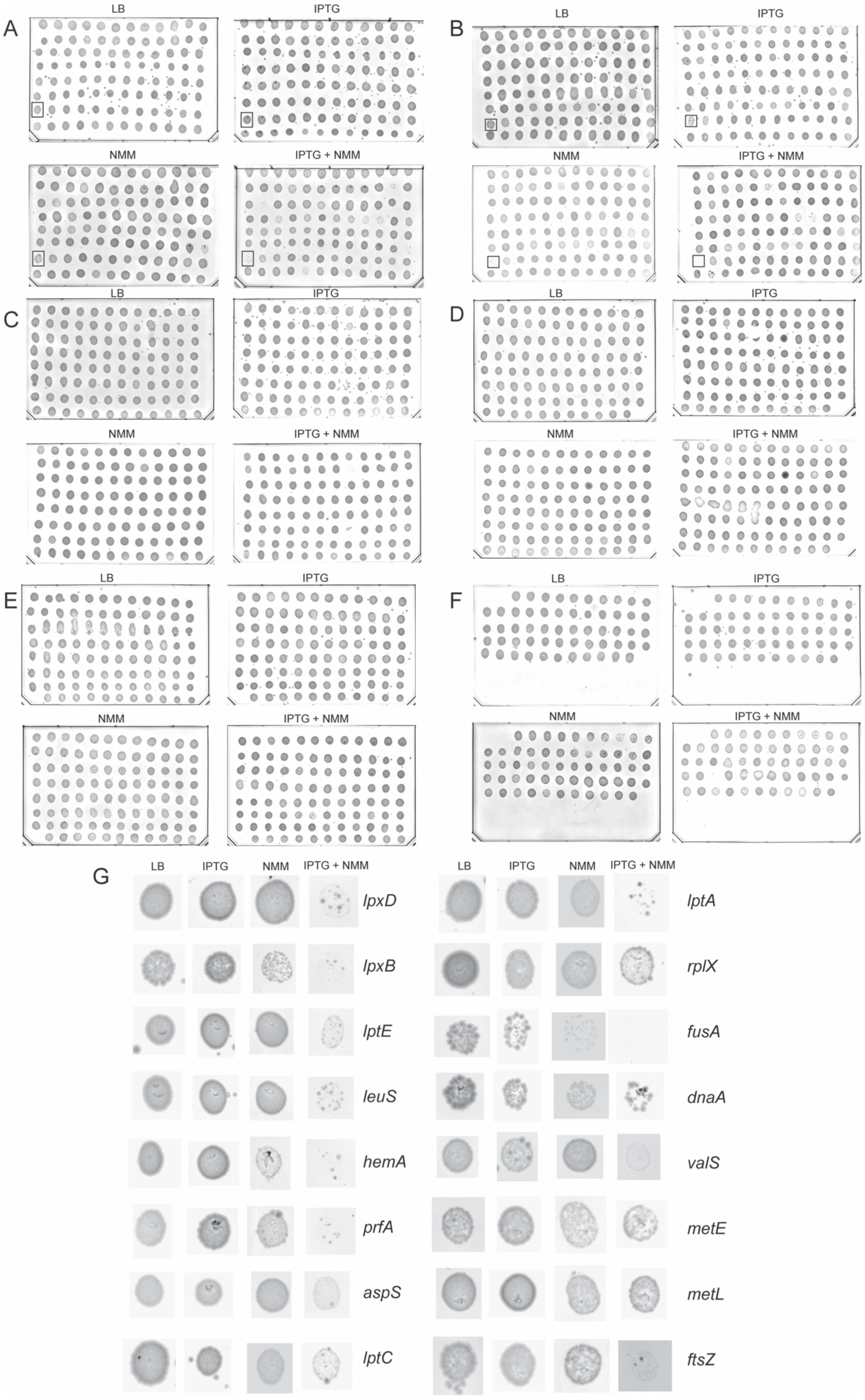
A
LB
IPTG
NMM
IPTG + NMM
B
LB
IPTG
NMM
IPTG + NMM
C
LB
IPTG
NMM
IPTG + NMM
D
LB
IPTG
NMM
IPTG + NMM
E
LB
IPTG
NMM
IPTG + NMM
F
LB
IPTG
NMM
IPTG + NMM
G
LB
IPTG
NMM
IPTG + NMM
lpxD
lpxB
lptE
leuS
hemA
prfA
aspS
lptC
LB
IPTG
NMM
IPTG + NMM
lptA
rplX
fusA
dnaA
valS
metE
metL
ftsZ

Figure S3: CRISPR interference plates testing sensitivity of knockdown strains to G4 stabilizer. (A-F). Test of each CRISPR interference strain for sensitivity to NMM, IPTG, and NMM IPTG and NMM together. The *prfA* knockdown strain is boxed in (A) and the *fusA* knockdown is boxed in (B). **(G).** Enlargement of spots for 16 gene knockdowns that had an impact on growth in NMM-treated conditions.

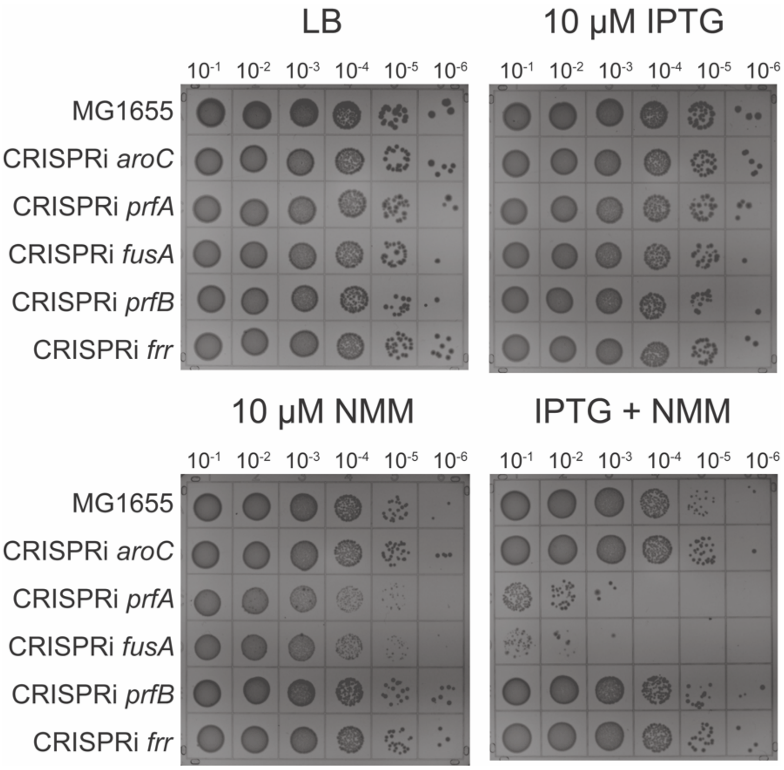

Figure S4: CRISPR interference machinery targeting *prfA* and *fusA* are sensitive to NMM. Spot dilution plates showing CRISPR interference machinery targeted to various genes. Spot plates contain LB-agar alone or LB-agar supplemented with IPTG, NMM, or both IPTG and NMM.

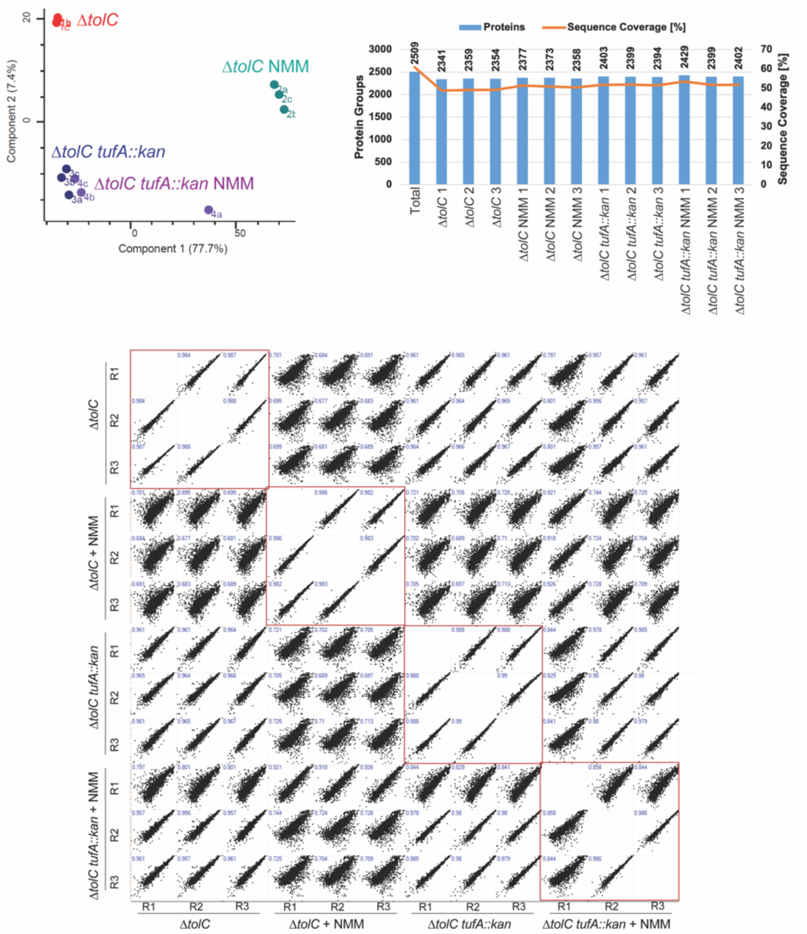

Figure S5: Proteomics quality control. Top left: principal component analysis of Δ*tolC,* Δ*tolC* NMM, Δ*tolC tufA::kan,* and Δ*tolC tufA::kan* NMM. Top right: total protein and sequence coverage for each replicate of the proteomics dataset. Bottom: Pearson correlation coefficients of each replicate/growth condition. Boxed in red are plots for correlation between replicates of each growth condition.

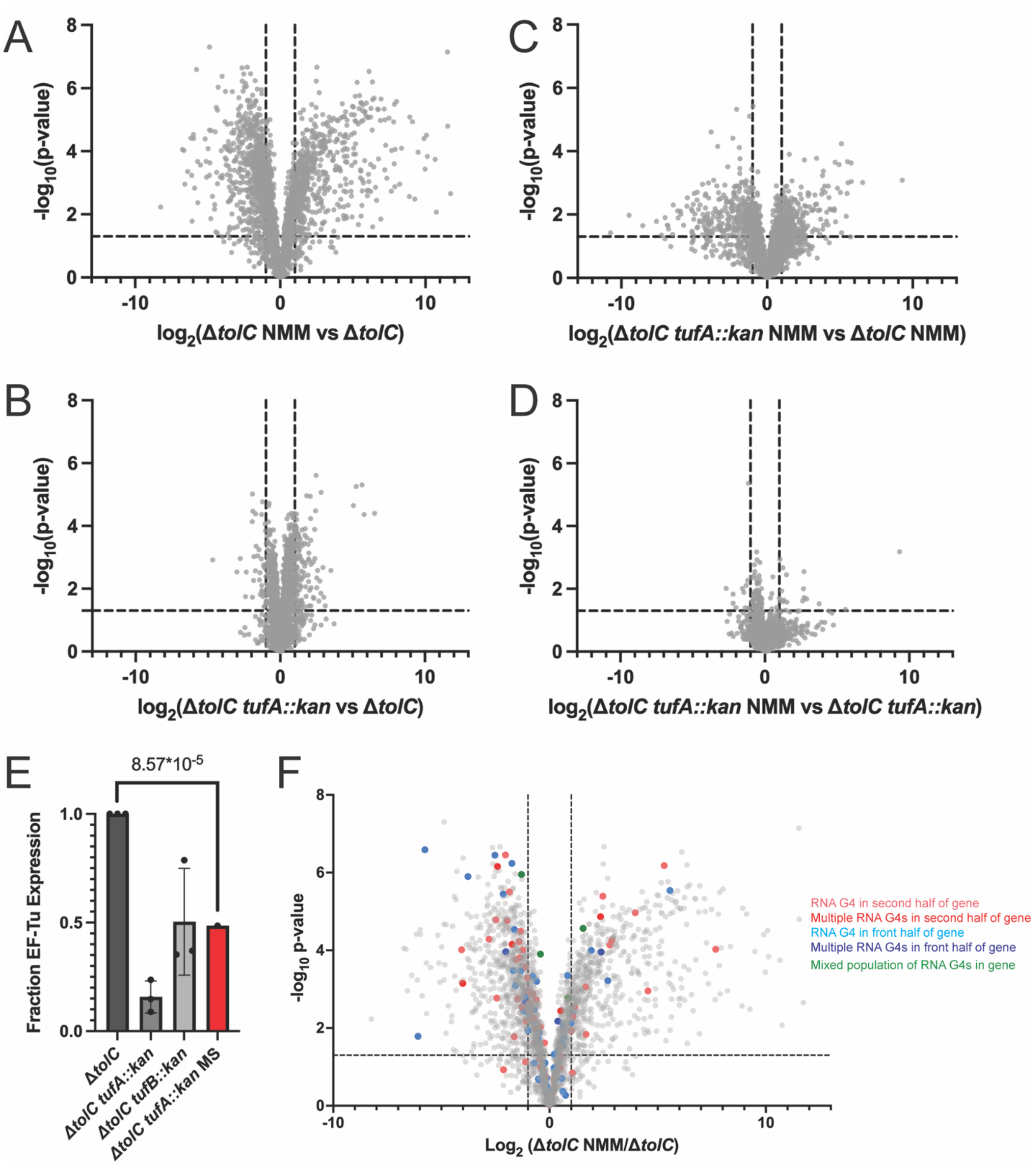

Figure S6: Proteomics dataset contains largest differences in ∆*tolC* NMM vs ∆*tolC* cells. (A-D). Volcano plots comparing proteomic landscape of different growth conditions. Horizontal dashed line indicates p-value of 0.05, and vertical lines indicate 2-

fold change in protein detection. Each dot represents a different protein detected in the dataset. **(E)**. Decrease in EF-Tu levels detected via mass spectrometry shown in red compared to what was detected via western blotting. **(F).** Location of RNA G4s in ORF mapped onto Δ*tolC* ± NMM Volcano plot.

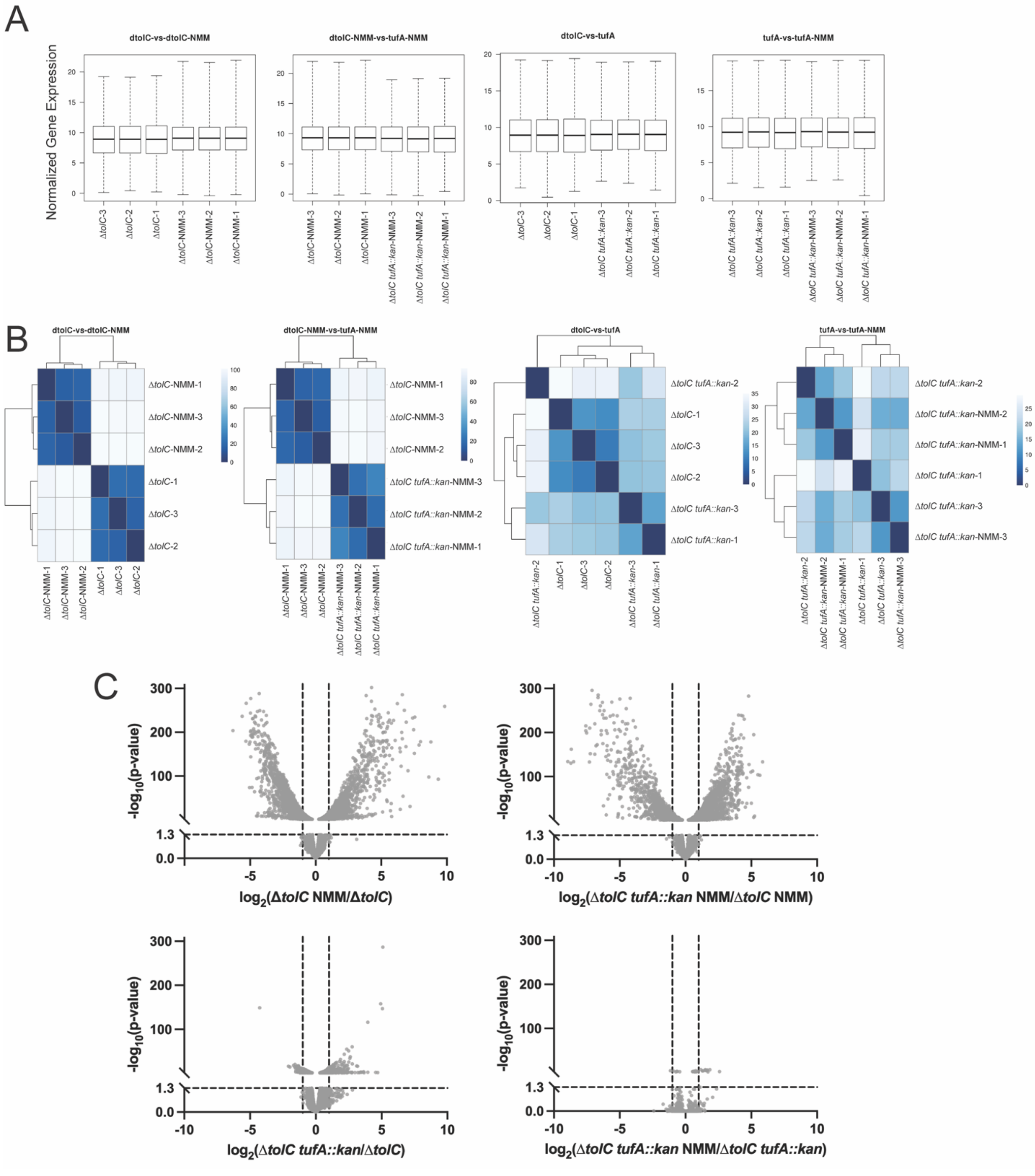

Figure S7: RNA-seq results. (A). Box and whisker plots of normalized gene expression for each growth condition. **(B)**. Sample distance plots generated using DeSeq2. Darker blue indicates closer correlation of expression values between

samples. **(C).** Volcano plots of log_2(Fold Change between normalized reads) for each transcript. Each y-axis had a break at p-value 0.05 to indicate significance and contains vertical lines at a two-fold change in detection of the transcript.

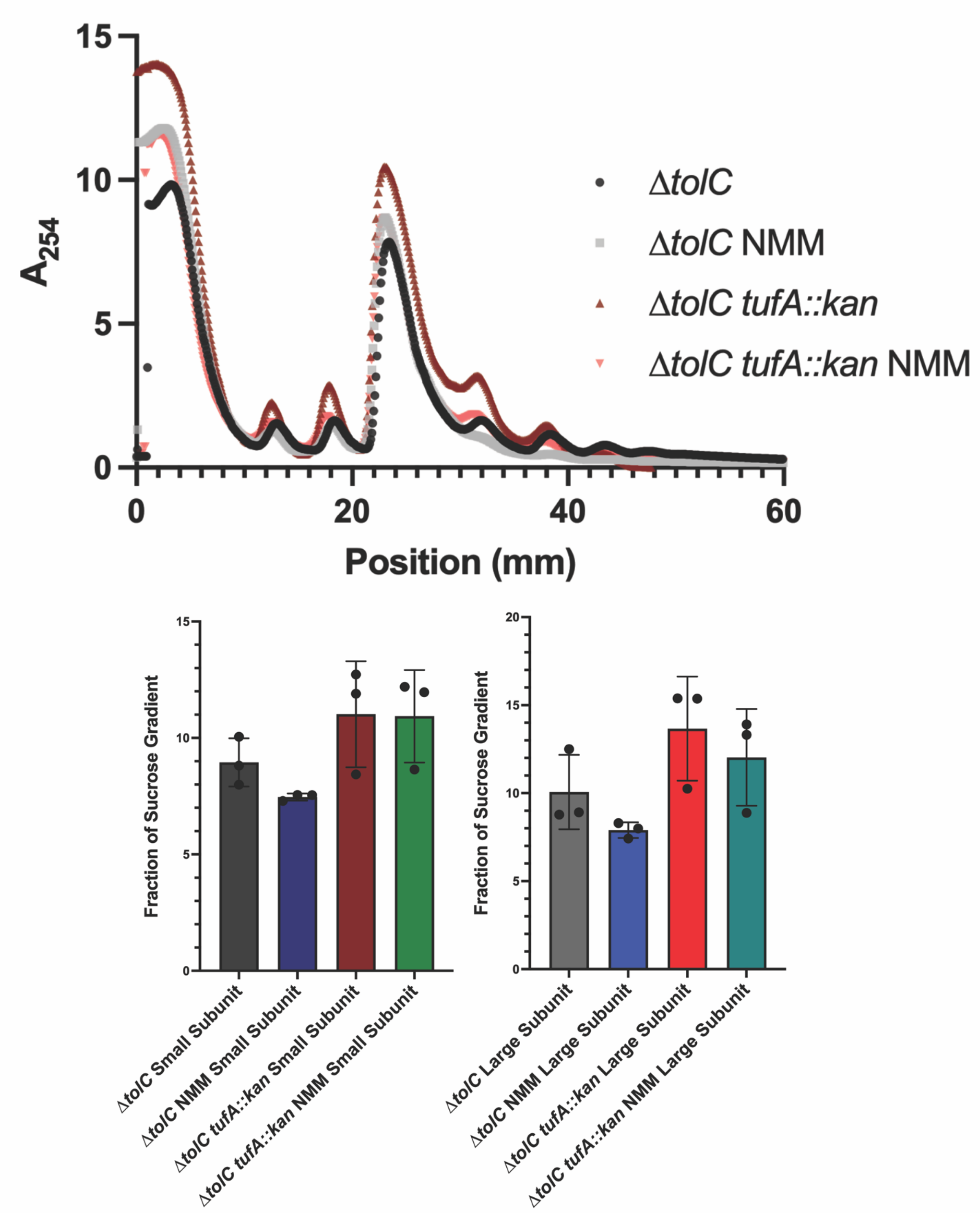

15
10
5
0
A254
0
20
40
60
Position (mm)
ΔtolC
ΔtolC NMM
ΔtolC tufA::kan
ΔtolC tufA::kan NMM
15
10
5
0
Fraction of Sucrose Gradient
ΔtolC Small Subunit
ΔtolC NMM Small Subunit
ΔtolC tufA::kan Small Subunit
ΔtolC tufA::kan NMM Small Subunit
20
15
10
5
0
Fraction of Sucrose Gradient
ΔtolC Large Subunit
ΔtolC NMM Large Subunit
ΔtolC tufA::kan Large Subunit
ΔtolC tufA::kan NMM Large Subunit

Figure S8: **Sucrose gradients separating ribosome components reveals differences in ∆*tolC* NMM monosome and polysome abundance**. Top: Representative sucrose gradients of each growth condition. Bottom: quantification of area under the curve for small subunit and large subunit. P-values that were significant via Welch's t-test are displayed.

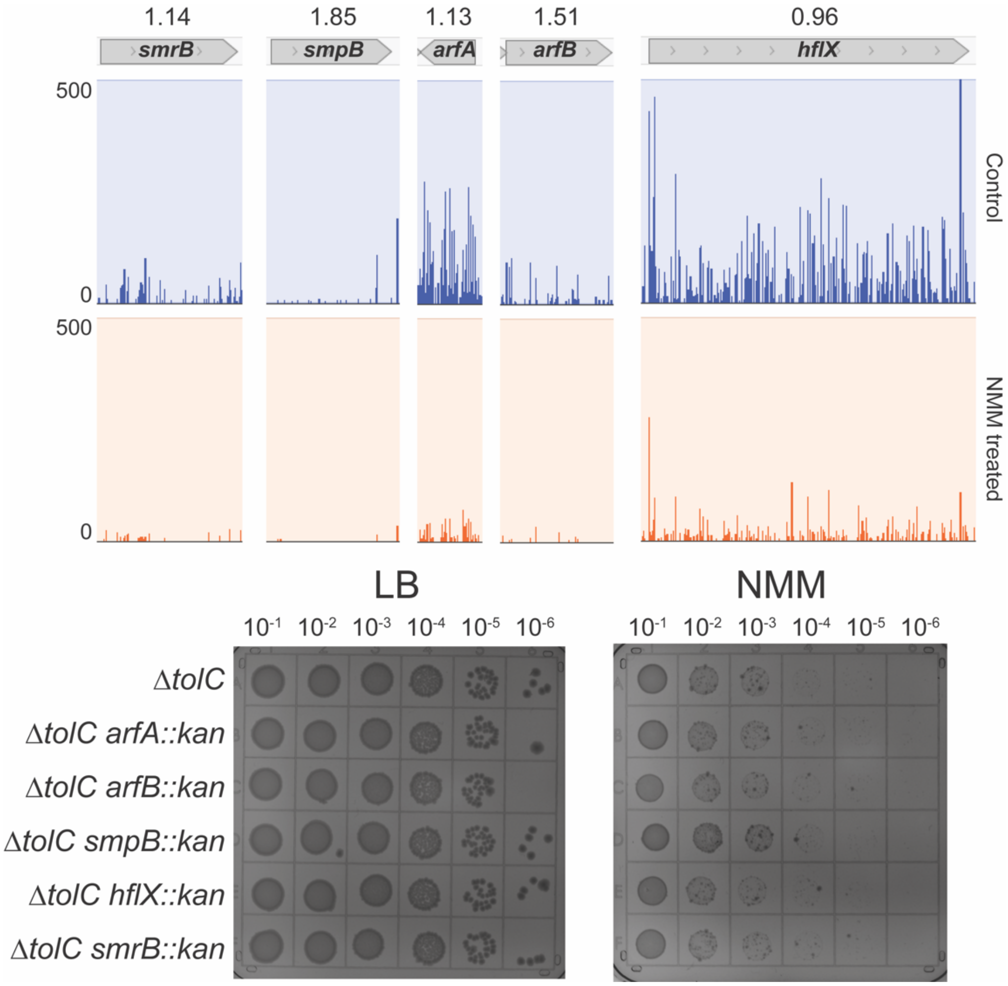

Figure S9: Ribosome rescue factors in Tn-seq screen and spot plates. Top: Mochiview plots of transposon insertions across various ribosome rescue factors. Log_{10}(ratio weighted reads) are above each Mochiview plot for each gene. Bottom: Spot plates of strains harboring ribosome rescue factor deletions in the presence and absence of NMM.

Supplementary Tables

Table S1: Gene ontology analysis of Tn-seq hits for which insertions are negatively selected in G4 stabilizing conditions

Gene ontology biological process	Fold Enrichment	p-value (FDR correction)
heme transport (GO:0015886)	2.56	1.40E-02
molybdopterin cofactor metabolic process (GO:0043545)	2.38	3.52E-02
Mo-molybdopterin cofactor biosynthetic process (GO:0006777)	2.38	3.45E-02
molybdopterin cofactor biosynthetic process (GO:0032324)	2.38	3.39E-02
Mo-molybdopterin cofactor metabolic process (GO:0019720)	2.38	3.32E-02
iron ion transmembrane transport (GO:0034755)	2.36	8.27E-04
IMP biosynthetic process (GO:0006188)	2.27	3.65E-02
rRNA modification (GO:0000154)	2.08	4.54E-03
cellular response to oxidative stress (GO:0034599)	1.89	4.10E-02
iron ion transport (GO:0006826)	1.87	2.90E-03
monosaccharide transmembrane transport (GO:0015749)	1.7	4.26E-02
transition metal ion transport (GO:0000041)	1.65	1.01E-02
cellular response to chemical stress (GO:0062197)	1.65	4.12E-02
metal ion transport (GO:0030001)	1.56	2.54E-03
proteinogenic amino acid biosynthetic process (GO:0170038)	1.53	3.60E-02
L-amino acid biosynthetic process (GO:0170034)	1.53	3.54E-02
amino acid biosynthetic process (GO:0008652)	1.46	1.31E-02

alpha-amino acid biosynthetic process (GO:1901607)	1.45	3.36E-02
monoatomic cation transmembrane transport (GO:0098655)	1.42	3.64E-02
inorganic cation transmembrane transport (GO:0098662)	1.42	3.91E-02
monoatomic cation transport (GO:0006812)	1.4	1.31E-02
monoatomic ion transmembrane transport (GO:0034220)	1.38	3.42E-02
DNA damage response (GO:0006974)	1.37	2.67E-03
monoatomic ion transport (GO:0006811)	1.34	2.75E-02
cellular response to stress (GO:0033554)	1.33	7.10E-04
regulation of RNA biosynthetic process (GO:2001141)	1.32	2.89E-03
regulation of DNA-templated transcription (GO:0006355)	1.32	2.65E-03
regulation of RNA metabolic process (GO:0051252)	1.3	3.60E-03
regulation of nucleobase-containing compound metabolic process (GO:0019219)	1.27	8.09E-03
response to stress (GO:0006950)	1.24	2.79E-03
cellular response to stimulus (GO:0051716)	1.2	3.24E-02
transport (GO:0006810)	1.17	3.41E-02
response to stimulus (GO:0050896)	1.16	7.08E-03
cellular component organization (GO:0016043)	0.77	1.55E-02
small molecule catabolic process (GO:0044282)	0.71	9.88E-03
generation of precursor metabolites and energy (GO:0006091)	0.69	4.61E-02
amide metabolic process (GO:0043603)	0.67	2.72E-02
lipid metabolic process (GO:0006629)	0.64	1.22E-02
monocarboxylic acid metabolic process (GO:0032787)	0.62	2.79E-03
organelle organization (GO:0006996)	0.57	1.58E-02
energy derivation by oxidation of organic compounds (GO:0015980)	0.56	9.97E-03

peptide metabolic process (GO:0006518)	0.55	6.45E-03
electron transport chain (GO:0022900)	0.54	1.57E-02
amide biosynthetic process (GO:0043604)	0.53	1.46E-03
cellular lipid metabolic process (GO:0044255)	0.49	3.29E-03
peptide biosynthetic process (GO:0043043)	0.44	5.78E-04
cell motility (GO:0048870)	0.43	3.69E-02
cellular respiration (GO:0045333)	0.39	3.29E-04
anaerobic respiration (GO:0009061)	0.36	9.87E-03
fatty acid metabolic process (GO:0006631)	0.36	7.23E-03
non-membrane-bounded organelle assembly (GO:0140694)	0.33	3.12E-03
organelle assembly (GO:0070925)	0.33	2.92E-03
anaerobic electron transport chain (GO:0019645)	0.33	3.61E-02
ribosome assembly (GO:0042255)	0.31	2.26E-02
oligosaccharide metabolic process (GO:0009311)	0.29	3.64E-02
translation (GO:0006412)	0.28	1.10E-06
respiratory electron transport chain (GO:0022904)	0.27	6.53E-04
protein-RNA complex organization (GO:0071826)	0.2	3.53E-03
protein-RNA complex assembly (GO:0022618)	0.2	3.37E-03
transposition (GO:0032196)	0.18	1.30E-04
DNA transposition (GO:0006313)	0.17	8.11E-04
ribosomal small subunit biogenesis (GO:0042274)	0.12	3.42E-02
nitrogen cycle metabolic process (GO:0071941)	0.1	1.04E-02
cytoplasmic translation (GO:0002181)	< 0.01	4.45E-07
reactive nitrogen species metabolic process (GO:2001057)	< 0.01	2.86E-03
response to cold (GO:0009409)	< 0.01	3.77E-02
nitrate assimilation (GO:0042128)	< 0.01	6.72E-03
nitrate metabolic process (GO:0042126)	< 0.01	6.48E-03

formate oxidation (GO:0015944)	< 0.01	3.71E-02
ribosomal small subunit assembly (GO:0000028)	< 0.01	2.94E-02

Table S2: Gene ontology analysis of Tn-seq hits for which insertions are positively selected in G4 stabilizing conditions

Gene ontology biological process	Fold Enrichment	p-value (FDR correction)
canonical glycolysis (GO:0061621)	35.49	2.05E-02
glycolytic process through glucose-6-phosphate (GO:0061620)	35.49	2.03E-02
lipoate biosynthetic process (GO:0009107)	35.49	2.01E-02
lipoate metabolic process (GO:0009106)	35.49	1.99E-02
regulation of glycogen metabolic process (GO:0070873)	35.49	1.98E-02
acetate biosynthetic process (GO:0019413)	35.49	1.96E-02
regulation of generation of precursor metabolites and energy (GO:0043467)	35.49	1.94E-02
NADH regeneration (GO:0006735)	35.49	1.93E-02
nitrogen fixation (GO:0009399)	35.49	1.91E-02
negative regulation of carbohydrate metabolic process (GO:0045912)	35.49	1.90E-02
glucose catabolic process to pyruvate (GO:0061718)	35.49	1.88E-02
tRNA wobble position uridine thiolation (GO:0002143)	30.42	6.42E-07

acetyl-CoA biosynthetic process (GO:0006085)	26.62	2.78E-03
glycolytic process through fructose-6-phosphate (GO:0061615)	23.66	4.52E-02
tRNA thio-modification (GO:0034227)	23.66	6.43E-06
lipid translocation (GO:0034204)	23.66	4.49E-02
regulation of membrane lipid distribution (GO:0097035)	23.66	4.46E-02
NAD catabolic process (GO:0019677)	23.66	4.43E-02
NADH metabolic process (GO:0006734)	21.3	6.39E-03
regulation of response to nutrient levels (GO:0032107)	17.75	1.11E-02
regulation of response to extracellular stimulus (GO:0032104)	17.75	1.10E-02
ribosomal small subunit assembly (GO:0000028)	15.97	2.47E-07
ribosomal small subunit biogenesis (GO:0042274)	13.65	1.96E-07
thioester biosynthetic process (GO:0035384)	13.31	2.47E-02
acyl-CoA biosynthetic process (GO:0071616)	13.31	2.45E-02
ribonucleoside diphosphate catabolic process (GO:0009191)	13.08	5.15E-05
purine ribonucleoside diphosphate catabolic process (GO:0009181)	13.08	4.96E-05
ADP catabolic process (GO:0046032)	13.08	4.79E-05
glycolytic process (GO:0006096)	13.08	4.62E-05
purine nucleoside diphosphate catabolic process (GO:0009137)	12.42	5.92E-05

nucleoside diphosphate catabolic process (GO:0009134)	12.42	5.75E-05
pyridine nucleotide catabolic process (GO:0019364)	12.42	5.59E-05
ADP metabolic process (GO:0046031)	12.42	5.44E-05
purine ribonucleoside diphosphate metabolic process (GO:0009179)	11.83	7.03E-05
purine ribonucleotide catabolic process (GO:0009154)	11.83	6.86E-05
ubiquinone biosynthetic process (GO:0006744)	11.83	6.71E-05
ubiquinone metabolic process (GO:0006743)	11.83	6.56E-05
acetyl-CoA metabolic process (GO:0006084)	11.83	3.38E-02
pyridine-containing compound catabolic process (GO:0072526)	11.29	8.81E-05
purine nucleoside diphosphate metabolic process (GO:0009135)	11.29	8.63E-05
tRNA wobble uridine modification (GO:0002098)	11.21	4.06E-04
ribonucleotide catabolic process (GO:0009261)	10.35	1.54E-04
purine nucleotide catabolic process (GO:0006195)	9.94	2.06E-04
ribonucleoside diphosphate metabolic process (GO:0009185)	9.56	2.47E-04
quinone biosynthetic process (GO:1901663)	8.87	4.11E-04
quinone metabolic process (GO:1901661)	8.87	4.05E-04
ATP metabolic process (GO:0046034)	8.87	3.98E-04

glyceraldehyde-3-phosphate metabolic process (GO:0019682)	8.87	1.97E-02
ketone biosynthetic process (GO:0042181)	8.57	4.90E-04
purine ribonucleoside triphosphate metabolic process (GO:0009205)	8.28	5.97E-04
tRNA wobble base modification (GO:0002097)	8.19	2.16E-03
nucleoside diphosphate metabolic process (GO:0009132)	8.01	7.43E-04
pyruvate metabolic process (GO:0006090)	7.76	8.46E-04
nicotinamide nucleotide metabolic process (GO:0046496)	7.55	4.63E-05
nucleotide catabolic process (GO:0009166)	7.53	1.04E-03
mRNA metabolic process (GO:0016071)	7.47	3.44E-02
ribonucleoside triphosphate metabolic process (GO:0009199)	7.31	1.21E-03
purine nucleoside triphosphate metabolic process (GO:0009144)	7.31	1.20E-03
nucleoside phosphate catabolic process (GO:1901292)	7.31	1.18E-03
pyridine nucleotide metabolic process (GO:0019362)	7.24	5.53E-05
ribosome assembly (GO:0042255)	7.24	5.39E-05
protein-RNA complex organization (GO:0071826)	6.94	2.06E-04
protein-RNA complex assembly (GO:0022618)	6.94	2.02E-04
negative regulation of translation (GO:0017148)	6.83	1.75E-02
purine-containing compound catabolic process (GO:0072523)	6.6	8.14E-04

nucleoside triphosphate metabolic process (GO:0009141)	6.6	8.03E-04
negative regulation of amide metabolic process (GO:0034249)	6.57	1.90E-02
pyridine-containing compound metabolic process (GO:0072524)	6.51	5.43E-05
negative regulation of gene expression (GO:0010629)	6.14	4.97E-04
nitrogen cycle metabolic process (GO:0071941)	5.92	2.89E-02
negative regulation of protein metabolic process (GO:0051248)	5.72	3.34E-02
positive regulation of gene expression (GO:0010628)	5.72	3.32E-02
non-membrane-bounded organelle assembly (GO:0140694)	5.35	2.35E-04
organelle assembly (GO:0070925)	5.35	2.31E-04
nucleobase-containing compound catabolic process (GO:0034655)	5.32	1.01E-04
ribosome biogenesis (GO:0042254)	5.27	1.51E-05
ribonucleoprotein complex biogenesis (GO:0022613)	5.22	1.56E-05
cellular ketone metabolic process (GO:0042180)	5.18	9.27E-03
sulfur compound biosynthetic process (GO:0044272)	5.07	8.20E-04
cytoplasmic translation (GO:0002181)	4.98	5.23E-03
post-transcriptional regulation of gene expression (GO:0010608)	4.91	2.35E-03
tRNA processing (GO:0008033)	4.86	1.12E-03
regulation of translation (GO:0006417)	4.81	6.35E-03
tRNA modification (GO:0006400)	4.81	6.29E-03

regulation of amide metabolic process (GO:0034248)	4.73	6.88E-03
translation (GO:0006412)	4.73	2.22E-05
peptide biosynthetic process (GO:0043043)	4.57	1.63E-05
organophosphate catabolic process (GO:0046434)	4.44	2.03E-02
tRNA metabolic process (GO:0006399)	4.26	7.74E-04
amide biosynthetic process (GO:0043604)	4.25	4.84E-06
purine nucleotide metabolic process (GO:0006163)	4.12	5.22E-04
purine ribonucleotide metabolic process (GO:0009150)	4.1	8.42E-03
peptide metabolic process (GO:0006518)	4.08	5.40E-05
RNA processing (GO:0006396)	3.91	8.28E-04
ncRNA processing (GO:0034470)	3.91	1.56E-03
regulation of protein metabolic process (GO:0051246)	3.84	2.29E-02
organelle organization (GO:0006996)	3.83	2.96E-04
purine-containing compound metabolic process (GO:0072521)	3.65	8.47E-04
ribose phosphate metabolic process (GO:0019693)	3.61	5.97E-03
ncRNA metabolic process (GO:0034660)	3.52	1.21E-03
RNA modification (GO:0009451)	3.47	2.29E-02
sulfur compound metabolic process (GO:0006790)	3.42	8.82E-03
ribonucleotide metabolic process (GO:0009259)	3.36	2.82E-02
amide metabolic process (GO:0043603)	3.33	8.86E-05

heterocycle catabolic process (GO:0046700)	3.33	6.44E-03
protein-containing complex assembly (GO:0065003)	3.33	1.23E-03
carbohydrate derivative catabolic process (GO:1901136)	3.3	5.00E-02
cellular nitrogen compound catabolic process (GO:0044270)	3.3	6.86E-03
gene expression (GO:0010467)	3.27	9.52E-09
protein-containing complex organization (GO:0043933)	3.1	2.60E-03
RNA metabolic process (GO:0016070)	3.08	6.18E-05
nucleotide metabolic process (GO:0009117)	3.01	5.82E-03
aromatic compound catabolic process (GO:0019439)	2.98	1.62E-02
nucleoside phosphate metabolic process (GO:0006753)	2.94	6.91E-03
small molecule biosynthetic process (GO:0044283)	2.86	6.67E-05
macromolecule modification (GO:0043412)	2.86	2.02E-02
macromolecule biosynthetic process (GO:0009059)	2.84	3.57E-10
positive regulation of cellular metabolic process (GO:0031325)	2.79	3.62E-02
positive regulation of macromolecule metabolic process (GO:0010604)	2.71	4.41E-02
organic cyclic compound catabolic process (GO:1901361)	2.66	3.40E-02
generation of precursor metabolites and energy (GO:0006091)	2.57	1.62E-02
cellular biosynthetic process (GO:0044249)	2.51	4.41E-15

organic substance biosynthetic process (GO:1901576)	2.51	2.77E-15
protein metabolic process (GO:0019538)	2.49	5.53E-04
negative regulation of metabolic process (GO:0009892)	2.49	2.03E-02
biosynthetic process (GO:0009058)	2.48	2.96E-15
cellular nitrogen compound biosynthetic process (GO:0044271)	2.44	5.33E-05
negative regulation of cellular metabolic process (GO:0031324)	2.38	4.05E-02
negative regulation of macromolecule metabolic process (GO:0010605)	2.34	4.51E-02
nucleobase-containing small molecule metabolic process (GO:0055086)	2.3	4.12E-02
organonitrogen compound biosynthetic process (GO:1901566)	2.26	1.28E-04
cellular component biogenesis (GO:0044085)	2.24	5.81E-03
organophosphate metabolic process (GO:0019637)	2.16	3.32E-02
cellular nitrogen compound metabolic process (GO:0034641)	2.12	1.97E-07
phosphate-containing compound metabolic process (GO:0006796)	2.07	9.02E-03
phosphorus metabolic process (GO:0006793)	2.04	1.11E-02
cellular component organization or biogenesis (GO:0071840)	2.01	3.99E-03
nucleobase-containing compound metabolic process (GO:0006139)	2	2.09E-04

organic cyclic compound biosynthetic process (GO:1901362)	2	3.39E-02
heterocycle biosynthetic process (GO:0018130)	1.99	4.42E-02
heterocycle metabolic process (GO:0046483)	1.95	6.58E-05
macromolecule metabolic process (GO:0043170)	1.94	1.55E-05
organonitrogen compound metabolic process (GO:1901564)	1.94	2.55E-05
carbohydrate derivative metabolic process (GO:1901135)	1.93	4.47E-02
organic cyclic compound metabolic process (GO:1901360)	1.89	9.88E-05
nitrogen compound metabolic process (GO:0006807)	1.84	1.98E-08
regulation of metabolic process (GO:0019222)	1.84	4.01E-02
nucleic acid metabolic process (GO:0090304)	1.82	4.41E-02
cellular aromatic compound metabolic process (GO:0006725)	1.76	1.89E-03
cellular metabolic process (GO:0044237)	1.74	6.38E-11
primary metabolic process (GO:0044238)	1.73	1.14E-07
biological regulation (GO:0065007)	1.69	3.41E-02
metabolic process (GO:0008152)	1.65	6.49E-11
organic substance metabolic process (GO:0071704)	1.65	1.66E-08
small molecule metabolic process (GO:0044281)	1.6	2.89E-02
cellular process (GO:0009987)	1.31	2.62E-05
biological_process (GO:0008150)	1.23	2.58E-05
Unclassified (UNCLASSIFIED)	0.24	2.48E-05

Table S3: Gene ontology analysis of proteins less abundant in G4 stabilizing conditions

Gene ontology biological process	Fold Enrichment	p-value (FDR correction)
ribosomal large subunit assembly (GO:0000027)	6.47	5.15E-08
ribosomal large subunit biogenesis (GO:0042273)	6.22	8.54E-08
cytoplasmic translation (GO:0002181)	6.03	1.84E-16
protein-RNA complex organization (GO:0071826)	6.01	4.14E-13
protein-RNA complex assembly (GO:0022618)	6.01	4.00E-13
ribosome assembly (GO:0042255)	5.91	1.35E-13
ribosomal small subunit assembly (GO:0000028)	5.73	4.05E-05
bacteriocin transport (GO:0043213)	5.39	2.57E-02
regulation of DNA-templated transcription elongation (GO:0032784)	4.9	3.65E-02
ribosomal small subunit biogenesis (GO:0042274)	4.67	1.44E-04
ribosome biogenesis (GO:0042254)	4.54	1.81E-17
ribonucleoprotein complex biogenesis (GO:0022613)	4.49	2.52E-17
amino acid activation (GO:0043038)	4.41	4.37E-04
non-membrane-bounded organelle assembly (GO:0140694)	4.34	1.76E-11
organelle assembly (GO:0070925)	4.34	1.71E-11
translation (GO:0006412)	4.32	6.99E-19
tRNA aminoacylation for protein translation (GO:0006418)	4.31	8.91E-04
lipopolysaccharide core region metabolic process (GO:0046401)	4.31	8.80E-04
lipopolysaccharide core region biosynthetic process (GO:0009244)	4.31	8.68E-04
tRNA aminoacylation (GO:0043039)	4.31	8.57E-04
peptide biosynthetic process (GO:0043043)	4.14	5.10E-19

DNA conformation change (GO:0071103)	4.13	2.71E-04
DNA-templated DNA replication maintenance of fidelity (GO:0045005)	4.04	4.75E-02
peptide metabolic process (GO:0006518)	3.78	7.45E-18
oligosaccharide biosynthetic process (GO:0009312)	3.72	2.82E-03
DNA duplex unwinding (GO:0032508)	3.65	1.26E-02
DNA geometric change (GO:0032392)	3.65	1.25E-02
amide biosynthetic process (GO:0043604)	3.59	3.92E-18
rRNA modification (GO:0000154)	3.58	2.46E-03
tRNA modification (GO:0006400)	3.54	5.44E-06
organelle organization (GO:0006996)	3.54	1.61E-14
rRNA methylation (GO:0031167)	3.52	2.51E-02
tRNA metabolic process (GO:0006399)	3.5	4.15E-10
DNA-templated DNA replication (GO:0006261)	3.46	1.38E-03
RNA modification (GO:0009451)	3.44	6.31E-09
tRNA processing (GO:0008033)	3.42	7.08E-07
RNA methylation (GO:0001510)	3.37	1.22E-03
negative regulation of translation (GO:0017148)	3.37	2.17E-02
DNA replication (GO:0006260)	3.31	3.90E-05
ncRNA metabolic process (GO:0034660)	3.3	1.45E-12
rRNA processing (GO:0006364)	3.29	7.51E-04
ncRNA processing (GO:0034470)	3.28	1.75E-09
negative regulation of amide metabolic process (GO:0034249)	3.25	2.71E-02
chromosome organization (GO:0051276)	3.24	4.16E-04
RNA processing (GO:0006396)	3.2	9.95E-10
macromolecule methylation (GO:0043414)	3.16	7.86E-04
rRNA metabolic process (GO:0016072)	3.15	1.14E-03
tRNA wobble base modification (GO:0002097)	3.11	4.95E-02
lipid localization (GO:0010876)	3.1	8.49E-03
lipid transport (GO:0006869)	3.1	8.40E-03
positive regulation of gene expression (GO:0010628)	3.04	2.81E-02
amide metabolic process (GO:0043603)	2.94	9.88E-15

establishment of localization in cell (GO:0051649)	2.63	4.81E-02
gene expression (GO:0010467)	2.62	1.14E-21
cell cycle process (GO:0022402)	2.6	8.76E-03
macromolecule modification (GO:0043412)	2.58	4.39E-07
methylation (GO:0032259)	2.53	8.46E-03
macromolecule biosynthetic process (GO:0009059)	2.5	1.77E-31
RNA metabolic process (GO:0016070)	2.49	5.65E-12
negative regulation of gene expression (GO:0010629)	2.46	4.30E-02
lipopolysaccharide biosynthetic process (GO:0009103)	2.43	6.52E-03
response to radiation (GO:0009314)	2.43	7.46E-04
cell cycle (GO:0007049)	2.42	1.62E-03
regulation of translation (GO:0006417)	2.4	2.82E-02
regulation of amide metabolic process (GO:0034248)	2.36	3.08E-02
cellular nitrogen compound biosynthetic process (GO:0044271)	2.36	1.64E-19
cell division (GO:0051301)	2.35	5.12E-03
lipopolysaccharide metabolic process (GO:0008653)	2.34	1.20E-02
protein-containing complex organization (GO:0043933)	2.31	5.42E-06
response to antibiotic (GO:0046677)	2.29	1.98E-03
post-transcriptional regulation of gene expression (GO:0010608)	2.28	4.10E-02
protein-containing complex assembly (GO:0065003)	2.27	3.06E-05
polysaccharide biosynthetic process (GO:0000271)	2.27	1.21E-03
carbohydrate derivative biosynthetic process (GO:1901137)	2.24	3.05E-08
protein metabolic process (GO:0019538)	2.22	1.79E-11
organonitrogen compound biosynthetic process (GO:1901566)	2.19	1.20E-17
cellular component biogenesis (GO:0044085)	2.16	5.01E-11
liposaccharide metabolic process (GO:1903509)	2.14	2.49E-02

polysaccharide metabolic process (GO:0005976)	2.1	3.81E-03
cellular biosynthetic process (GO:0044249)	2.04	3.07E-33
nucleotide biosynthetic process (GO:0009165)	2.04	2.97E-02
nucleoside phosphate biosynthetic process (GO:1901293)	2.04	2.94E-02
cellular component organization or biogenesis (GO:0071840)	2.03	1.38E-13
regulation of biological quality (GO:0065008)	2.03	2.50E-02
organic substance biosynthetic process (GO:1901576)	2.02	1.69E-33
biosynthetic process (GO:0009058)	1.99	7.70E-33
lipid biosynthetic process (GO:0008610)	1.99	5.60E-03
nucleic acid metabolic process (GO:0090304)	1.98	7.17E-12
macromolecule metabolic process (GO:0043170)	1.98	2.87E-28
carbohydrate biosynthetic process (GO:0016051)	1.96	1.25E-02
nucleobase-containing compound biosynthetic process (GO:0034654)	1.95	5.29E-05
macromolecule localization (GO:0033036)	1.93	8.81E-03
cellular component assembly (GO:0022607)	1.92	4.39E-05
cellular component organization (GO:0016043)	1.91	1.75E-09
cellular nitrogen compound metabolic process (GO:0034641)	1.88	5.58E-22
nucleobase-containing compound metabolic process (GO:0006139)	1.78	2.34E-12
organophosphate biosynthetic process (GO:0090407)	1.76	1.18E-02
aromatic compound biosynthetic process (GO:0019438)	1.76	1.40E-04
response to abiotic stimulus (GO:0009628)	1.68	5.44E-03
organic cyclic compound biosynthetic process (GO:1901362)	1.67	4.85E-04
nitrogen compound transport (GO:0071705)	1.67	6.30E-03
heterocycle biosynthetic process (GO:0018130)	1.66	8.66E-04
heterocycle metabolic process (GO:0046483)	1.63	2.97E-10

carbohydrate derivative metabolic process (GO:1901135)	1.6	1.14E-03
cellular aromatic compound metabolic process (GO:0006725)	1.6	1.87E-09
organic cyclic compound metabolic process (GO:1901360)	1.57	3.31E-09
nitrogen compound metabolic process (GO:0006807)	1.55	2.73E-17
primary metabolic process (GO:0044238)	1.49	1.47E-16
organonitrogen compound metabolic process (GO:1901564)	1.47	7.98E-07
cellular metabolic process (GO:0044237)	1.42	2.52E-16
organic substance metabolic process (GO:0071704)	1.38	7.79E-14
metabolic process (GO:0008152)	1.32	2.24E-12
biological regulation (GO:0065007)	1.31	4.98E-02
cellular process (GO:0009987)	1.28	3.40E-22
biological_process (GO:0008150)	1.19	1.15E-18
cellular catabolic process (GO:0044248)	0.48	1.32E-03
monocarboxylic acid metabolic process (GO:0032787)	0.48	3.57E-02
organonitrogen compound catabolic process (GO:1901565)	0.48	2.82E-02
organic substance catabolic process (GO:1901575)	0.47	2.04E-05
catabolic process (GO:0009056)	0.45	3.77E-06
carboxylic acid catabolic process (GO:0046395)	0.37	6.74E-03
Unclassified (UNCLASSIFIED)	0.36	1.06E-18
organic acid catabolic process (GO:0016054)	0.35	2.86E-03
small molecule catabolic process (GO:0044282)	0.29	1.77E-06
carbohydrate catabolic process (GO:0016052)	0.2	1.31E-03
monosaccharide metabolic process (GO:0005996)	0.12	1.13E-03
carbohydrate transport (GO:0008643)	0.1	1.87E-04
transposition (GO:0032196)	0.1	3.73E-02
hexose metabolic process (GO:0019318)	0.09	1.29E-02
carbohydrate transmembrane transport (GO:0034219)	0.06	2.32E-04

Table S4: Gene ontology analysis of proteins more abundant in G4 stabilizing conditions

Gene ontology biological process	Fold Enrichment	p-value (FDR correction)
glycogen metabolic process (GO:0005977)	8.08	1.32E-02
energy reserve metabolic process (GO:0006112)	8.08	1.30E-02
N-acetylneuraminate catabolic process (GO:0019262)	6.46	1.30E-02
alditol catabolic process (GO:0019405)	6.28	3.88E-04
ketone catabolic process (GO:0042182)	6.21	4.17E-03
glycerol catabolic process (GO:0019563)	6.06	9.76E-03
polyol catabolic process (GO:0046174)	6.06	2.61E-04
methylglyoxal metabolic process (GO:0009438)	5.87	1.78E-02
N-acetylneuraminate metabolic process (GO:0006054)	5.87	1.76E-02
methylglyoxal catabolic process (GO:0051596)	5.65	3.47E-02
polysaccharide catabolic process (GO:0000272)	5.38	2.28E-02
hexitol metabolic process (GO:0006059)	5.14	4.56E-02
oligosaccharide catabolic process (GO:0009313)	5.14	4.53E-02
dicarboxylic acid catabolic process (GO:0043649)	4.97	3.04E-02
disaccharide metabolic process (GO:0005984)	4.85	1.99E-02
amino sugar catabolic process (GO:0046348)	4.85	1.97E-02
ribonucleoside diphosphate catabolic process (GO:0009191)	4.68	9.96E-03

purine ribonucleoside diphosphate catabolic process (GO:0009181)	4.68	9.80E-03
aldehyde catabolic process (GO:0046185)	4.68	9.64E-03
ADP catabolic process (GO:0046032)	4.68	9.49E-03
glycolytic process (GO:0006096)	4.68	9.34E-03
purine nucleoside diphosphate catabolic process (GO:0009137)	4.44	1.23E-02
nucleoside diphosphate catabolic process (GO:0009134)	4.44	1.21E-02
pyridine nucleotide catabolic process (GO:0019364)	4.44	1.19E-02
ADP metabolic process (GO:0046031)	4.44	1.18E-02
purine ribonucleoside diphosphate metabolic process (GO:0009179)	4.23	1.49E-02
purine ribonucleotide catabolic process (GO:0009154)	4.23	1.47E-02
nucleotide catabolic process (GO:0009166)	4.16	1.45E-03
alditol metabolic process (GO:0019400)	4.16	1.40E-03
pyridine-containing compound catabolic process (GO:0072526)	4.04	1.75E-02
purine nucleoside diphosphate metabolic process (GO:0009135)	4.04	1.73E-02
pyruvate metabolic process (GO:0006090)	4.04	2.02E-03
nucleoside phosphate catabolic process (GO:1901292)	4.04	1.44E-03
polyol metabolic process (GO:0019751)	3.93	1.37E-03
purine nucleotide catabolic process (GO:0006195)	3.88	1.45E-02
ribonucleotide catabolic process (GO:0009261)	3.7	2.60E-02
glycerol metabolic process (GO:0006071)	3.67	3.93E-02
cellular oxidant detoxification (GO:0098869)	3.51	4.72E-02

carbohydrate catabolic process (GO:0016052)	3.51	3.30E-11
carbohydrate derivative catabolic process (GO:1901136)	3.47	1.66E-06
ribonucleoside diphosphate metabolic process (GO:0009185)	3.42	3.77E-02
organophosphate catabolic process (GO:0046434)	3.32	1.05E-03
alcohol catabolic process (GO:0046164)	3.16	6.72E-03
cellular response to oxidative stress (GO:0034599)	3.03	4.76E-02
nicotinamide nucleotide metabolic process (GO:0046496)	2.92	1.47E-02
response to reactive oxygen species (GO:0000302)	2.88	2.63E-02
pyridine nucleotide metabolic process (GO:0019362)	2.8	1.86E-02
organic hydroxy compound catabolic process (GO:1901616)	2.8	2.04E-02
cellular response to chemical stress (GO:0062197)	2.55	3.03E-02
response to oxidative stress (GO:0006979)	2.53	1.50E-03
response to oxygen-containing compound (GO:1901700)	2.35	1.80E-02
monosaccharide metabolic process (GO:0005996)	2.34	2.90E-03
amino acid catabolic process (GO:0009063)	2.32	2.12E-02
hexose metabolic process (GO:0019318)	2.31	2.74E-02
small molecule catabolic process (GO:0044282)	2.26	1.67E-08
protein complex oligomerization (GO:0051259)	2.25	4.15E-02
organic substance catabolic process (GO:1901575)	2.12	3.45E-12

catabolic process (GO:0009056)	2.12	2.23E-12
organonitrogen compound catabolic process (GO:1901565)	2.11	7.97E-05
carbohydrate transmembrane transport (GO:0034219)	2.11	2.37E-02
carbohydrate metabolic process (GO:0005975)	2.09	4.72E-08
cellular catabolic process (GO:0044248)	2.04	3.75E-07
carboxylic acid catabolic process (GO:0046395)	2.01	1.49E-03
carbohydrate transport (GO:0008643)	1.94	4.15E-02
organic hydroxy compound metabolic process (GO:1901615)	1.93	4.23E-02
organic acid catabolic process (GO:0016054)	1.9	3.85E-03
cellular response to stress (GO:0033554)	1.89	8.57E-06
DNA damage response (GO:0006974)	1.81	2.09E-03
monocarboxylic acid metabolic process (GO:0032787)	1.73	1.62E-02
response to stress (GO:0006950)	1.7	1.56E-05
oxoacid metabolic process (GO:0043436)	1.59	1.11E-03
carboxylic acid metabolic process (GO:0019752)	1.58	1.50E-03
cellular response to stimulus (GO:0051716)	1.56	1.42E-03
organic acid metabolic process (GO:0006082)	1.55	1.92E-03
small molecule metabolic process (GO:0044281)	1.49	8.12E-05
response to stimulus (GO:0050896)	1.27	3.79E-02
establishment of localization (GO:0051234)	0.69	3.94E-02
localization (GO:0051179)	0.69	3.43E-02
biosynthetic process (GO:0009058)	0.68	2.12E-03

organic substance biosynthetic process (GO:1901576)	0.67	1.44E-03
organic cyclic compound metabolic process (GO:1901360)	0.66	3.35E-03
heterocycle metabolic process (GO:0046483)	0.65	3.10E-03
regulation of cellular process (GO:0050794)	0.64	3.05E-02
macromolecule metabolic process (GO:0043170)	0.63	4.38E-04
cellular aromatic compound metabolic process (GO:0006725)	0.62	1.14E-03
cellular biosynthetic process (GO:0044249)	0.61	8.47E-05
regulation of primary metabolic process (GO:0080090)	0.6	4.15E-02
nucleobase-containing compound metabolic process (GO:0006139)	0.6	1.86E-03
regulation of nitrogen compound metabolic process (GO:0051171)	0.59	3.94E-02
regulation of metabolic process (GO:0019222)	0.59	2.32E-02
cellular nitrogen compound metabolic process (GO:0034641)	0.57	3.41E-05
regulation of nucleobase-containing compound metabolic process (GO:0019219)	0.57	4.39E-02
regulation of cellular metabolic process (GO:0031323)	0.57	1.79E-02
regulation of macromolecule metabolic process (GO:0060255)	0.57	1.74E-02
regulation of RNA metabolic process (GO:0051252)	0.55	3.14E-02
regulation of RNA biosynthetic process (GO:2001141)	0.55	3.46E-02
regulation of DNA-templated transcription (GO:0006355)	0.55	3.43E-02

organonitrogen compound biosynthetic process (GO:1901566)	0.55	3.79E-03
regulation of biosynthetic process (GO:0009889)	0.54	1.18E-02
regulation of gene expression (GO:0010468)	0.53	1.11E-02
regulation of macromolecule biosynthetic process (GO:0010556)	0.53	9.92E-03
regulation of cellular biosynthetic process (GO:0031326)	0.53	8.12E-03
gene expression (GO:0010467)	0.5	9.54E-03
macromolecule biosynthetic process (GO:0009059)	0.5	2.80E-04
organic cyclic compound biosynthetic process (GO:1901362)	0.48	8.06E-03
nitrogen compound transport (GO:0071705)	0.48	4.10E-02
heterocycle biosynthetic process (GO:0018130)	0.48	9.78E-03
carbohydrate derivative biosynthetic process (GO:1901137)	0.44	2.56E-02
nucleobase-containing compound biosynthetic process (GO:0034654)	0.39	1.06E-02
aromatic compound biosynthetic process (GO:0019438)	0.39	1.47E-03
cellular nitrogen compound biosynthetic process (GO:0044271)	0.39	3.01E-05
RNA metabolic process (GO:0016070)	0.34	1.95E-03
DNA metabolic process (GO:0006259)	0.33	1.52E-03
nucleic acid metabolic process (GO:0090304)	0.3	1.02E-07
lipid biosynthetic process (GO:0008610)	0.28	3.08E-02
macromolecule modification (GO:0043412)	0.27	1.90E-02
translation (GO:0006412)	0.2	2.01E-02
tRNA metabolic process (GO:0006399)	0.16	3.04E-02
RNA processing (GO:0006396)	0.14	8.72E-03

organelle organization (GO:0006996)	0.12	1.51E-03
ncRNA metabolic process (GO:0034660)	0.11	1.46E-03
cell projection organization (GO:0030030)	0.1	4.05E-02
cell division (GO:0051301)	0.1	3.07E-02
liposaccharide metabolic process (GO:1903509)	0.1	2.29E-02
RNA modification (GO:0009451)	0.09	1.36E-02
DNA recombination (GO:0006310)	0.07	1.45E-03
tRNA processing (GO:0008033)	< 0.01	1.56E-02
non-membrane-bounded organelle assembly (GO:0140694)	< 0.01	1.54E-02
organelle assembly (GO:0070925)	< 0.01	1.52E-02
proton transmembrane transport (GO:1902600)	< 0.01	2.60E-02
ribosome biogenesis (GO:0042254)	< 0.01	1.47E-03
ribonucleoprotein complex biogenesis (GO:0022613)	< 0.01	1.17E-03
ncRNA processing (GO:0034470)	< 0.01	9.22E-04
cell cycle process (GO:0022402)	< 0.01	4.76E-02
cell cycle (GO:0007049)	< 0.01	3.01E-03
transposition (GO:0032196)	< 0.01	1.91E-02

Table S5: Comparison of GO terms identified in proteomics (as proteins less abundant in G4 stabilizing conditions) and genes that harbored more insertions in G4 stabilizing conditions

**GO terms detected in Tn-seq (more transposon insertions in NMM) and GO terms detected in proteomics (as less abundant in NMM)**
ribosomal small subunit assembly (GO:0000028)
ribosomal small subunit biogenesis (GO:0042274)
tRNA wobble base modification (GO:0002097)
ribosome assembly (GO:0042255)
protein-RNA complex organization (GO:0071826)

protein-RNA complex assembly (GO:0022618)
negative regulation of translation (GO:0017148)
negative regulation of amide metabolic process (GO:0034249)
negative regulation of gene expression (GO:0010629)
positive regulation of gene expression (GO:0010628)
non-membrane-bounded organelle assembly (GO:0140694)
organelle assembly (GO:0070925)
ribosome biogenesis (GO:0042254)
ribonucleoprotein complex biogenesis (GO:0022613)
cytoplasmic translation (GO:0002181)
post-transcriptional regulation of gene expression (GO:0010608)
tRNA processing (GO:0008033)
regulation of translation (GO:0006417)
tRNA modification (GO:0006400)
regulation of amide metabolic process (GO:0034248)
translation (GO:0006412)
peptide biosynthetic process (GO:0043043)
tRNA metabolic process (GO:0006399)
amide biosynthetic process (GO:0043604)
peptide metabolic process (GO:0006518)
RNA processing (GO:0006396)
ncRNA processing (GO:0034470)
organelle organization (GO:0006996)
ncRNA metabolic process (GO:0034660)
RNA modification (GO:0009451)
amide metabolic process (GO:0043603)
protein-containing complex assembly (GO:0065003)
gene expression (GO:0010467)
protein-containing complex organization (GO:0043933)
RNA metabolic process (GO:0016070)
macromolecule modification (GO:0043412)
macromolecule biosynthetic process (GO:0009059)
cellular biosynthetic process (GO:0044249)
organic substance biosynthetic process (GO:1901576)
protein metabolic process (GO:0019538)
biosynthetic process (GO:0009058)
cellular nitrogen compound biosynthetic process (GO:0044271)
organonitrogen compound biosynthetic process (GO:1901566)
cellular component biogenesis (GO:0044085)

cellular nitrogen compound metabolic process (GO:0034641)
cellular component organization or biogenesis (GO:0071840)
nucleobase-containing compound metabolic process (GO:0006139)
organic cyclic compound biosynthetic process (GO:1901362)
heterocycle biosynthetic process (GO:0018130)
heterocycle metabolic process (GO:0046483)
macromolecule metabolic process (GO:0043170)
organonitrogen compound metabolic process (GO:1901564)
carbohydrate derivative metabolic process (GO:1901135)
organic cyclic compound metabolic process (GO:1901360)
nitrogen compound metabolic process (GO:0006807)
nucleic acid metabolic process (GO:0090304)
cellular aromatic compound metabolic process (GO:0006725)
cellular metabolic process (GO:0044237)
primary metabolic process (GO:0044238)
biological regulation (GO:0065007)
metabolic process (GO:0008152)
organic substance metabolic process (GO:0071704)
cellular process (GO:0009987)
biological process (GO:0008150)

Table S6: Upregulated proteins in ∆*tolC tufA::kan* compared to ∆*tolC*

Gene ontology biological process	Fold Enrichment	p-value (FDR correction)
alanine catabolic process (GO:0006524)	27.97	3.79E-02
leucine biosynthetic process (GO:0009098)	23.31	1.50E-03
leucine metabolic process (GO:0006551)	23.31	1.44E-03
valine biosynthetic process (GO:0009099)	20.34	3.85E-04
valine metabolic process (GO:0006573)	20.34	3.68E-04
isoleucine biosynthetic process (GO:0009097)	19.89	1.05E-05
isoleucine metabolic process (GO:0006549)	19.89	9.91E-06
branched-chain amino acid biosynthetic process (GO:0009082)	17.9	1.65E-08

branched-chain amino acid metabolic process (GO:0009081)	17.9	1.51E-08
indolalkylamine biosynthetic process (GO:0046219)	14.92	2.74E-02
indole-containing compound biosynthetic process (GO:0042435)	14.92	2.67E-02
tryptophan biosynthetic process (GO:0000162)	14.92	2.61E-02
methionine biosynthetic process (GO:0009086)	11.66	1.60E-02
biogenic amine biosynthetic process (GO:0042401)	11.66	1.55E-02
amine biosynthetic process (GO:0009309)	11.66	1.50E-02
methionine metabolic process (GO:0006555)	11.66	1.46E-02
indole-containing compound metabolic process (GO:0042430)	11.48	4.88E-02
indolalkylamine metabolic process (GO:0006586)	11.48	4.78E-02
tryptophan metabolic process (GO:0006568)	11.48	4.69E-02
alpha-amino acid biosynthetic process (GO:1901607)	9.25	7.08E-16
aromatic amino acid family biosynthetic process (GO:0009073)	8.61	1.48E-02
aspartate family amino acid biosynthetic process (GO:0009067)	8.29	1.57E-03
sulfur amino acid biosynthetic process (GO:0000097)	8.29	1.51E-02
amino acid biosynthetic process (GO:0008652)	8.2	1.64E-15
tricarboxylic acid cycle (GO:0006099)	7.46	2.29E-02
non-proteinogenic amino acid metabolic process (GO:0170041)	6.78	3.17E-02
aromatic amino acid metabolic process (GO:0009072)	6.78	3.10E-02

proteinogenic amino acid biosynthetic process (GO:0170038)	6.43	8.02E-06
L-amino acid biosynthetic process (GO:0170034)	6.43	7.48E-06
alpha-amino acid metabolic process (GO:1901605)	6.34	1.94E-14
dicarboxylic acid biosynthetic process (GO:0043650)	6.22	4.37E-02
sulfur amino acid metabolic process (GO:0000096)	6.05	4.86E-02
organic acid biosynthetic process (GO:0016053)	5.82	1.15E-12
carboxylic acid biosynthetic process (GO:0046394)	5.82	9.56E-13
aspartate family amino acid metabolic process (GO:0009066)	5.59	6.67E-03
amino acid metabolic process (GO:0006520)	5.48	8.86E-14
aerobic respiration (GO:0009060)	4.97	2.65E-02
sulfur compound biosynthetic process (GO:0044272)	4.8	1.56E-02
proteinogenic amino acid metabolic process (GO:0170039)	4.66	1.04E-05
L-amino acid metabolic process (GO:0170033)	4.63	9.68E-06
small molecule biosynthetic process (GO:0044283)	4.09	7.81E-10
carboxylic acid metabolic process (GO:0019752)	3.43	1.12E-11
oxoacid metabolic process (GO:0043436)	3.38	4.87E-12
organic acid metabolic process (GO:0006082)	3.3	1.11E-11
energy derivation by oxidation of organic compounds (GO:0015980)	3.17	3.72E-02
organonitrogen compound biosynthetic process (GO:1901566)	2.45	8.71E-05

small molecule metabolic process (GO:0044281)	2.19	2.47E-06
organonitrogen compound metabolic process (GO:1901564)	1.85	1.17E-03
biosynthetic process (GO:0009058)	1.58	4.77E-02
primary metabolic process (GO:0044238)	1.52	2.17E-03
nucleobase-containing compound metabolic process (GO:0006139)	0.3	1.85E-02

Table S7: Downregulated proteins in Δ*tolC tufA::kan* compared to Δ*tolC*

Gene ontology biological process	Fold enrichment	p-value (FDR correction)
'de novo' UMP biosynthetic process (GO:0044205)	60.84	2.66E-02
'de novo' pyrimidine nucleobase biosynthetic process (GO:0006207)	47.32	3.22E-02
pyrimidine ribonucleoside monophosphate biosynthetic process (GO:0009174)	42.59	3.58E-02
UMP biosynthetic process (GO:0006222)	42.59	3.13E-02
pyrimidine nucleobase biosynthetic process (GO:0019856)	40.56	1.66E-02
pyrimidine ribonucleoside monophosphate metabolic process (GO:0009173)	35.49	3.94E-02
UMP metabolic process (GO:0046049)	35.49	3.59E-02
pyrimidine ribonucleotide biosynthetic process (GO:0009220)	32.76	4.03E-02
nucleobase biosynthetic process (GO:0046112)	28.39	2.80E-02

pyrimidine nucleotide biosynthetic process (GO:0006221)	24.69	3.03E-02
pyrimidine nucleotide metabolic process (GO:0006220)	21.03	3.20E-02
pyrimidine nucleobase metabolic process (GO:0006206)	18.32	2.90E-02

Table S8: Proteins less abundant in Δ*tolC tufA::kan* NMM than Δ*tolC* NMM

Gene ontology biological process	Fold enrichment	p-value (FDR correction)
response to reactive oxygen species (GO:0000302)	4.47	3.11E-02
nicotinamide nucleotide metabolic process (GO:0046496)	4.36	2.58E-02
cellular response to chemical stress (GO:0062197)	4.19	1.06E-02
pyridine nucleotide metabolic process (GO:0019362)	4.18	2.91E-02
response to oxidative stress (GO:0006979)	3.68	2.34E-03
cellular response to stress (GO:0033554)	1.9	2.47E-02
response to stress (GO:0006950)	1.82	3.11E-03
transport (GO:0006810)	0.38	1.32E-03
establishment of localization (GO:0051234)	0.37	1.25E-03
localization (GO:0051179)	0.37	1.33E-03
transmembrane transport (GO:0055085)	0.35	2.59E-03

Table S9: Proteins more abundant in Δ*tolC tufA::kan* NMM than Δ*tolC* NMM

Gene ontology biological process	Fold enrichment	p-value (FDR correction)
leucine biosynthetic process (GO:0009098)	13.42	9.27E-03

leucine metabolic process (GO:0006551)	13.42	9.10E-03
tRNA aminoacylation for protein translation (GO:0006418)	7.73	8.67E-04
branched-chain amino acid biosynthetic process (GO:0009082)	7.73	8.46E-04
branched-chain amino acid metabolic process (GO:0009081)	7.73	8.25E-04
tRNA aminoacylation (GO:0043039)	7.73	8.06E-04
amino acid activation (GO:0043038)	7.43	9.73E-04
DNA biosynthetic process (GO:0071897)	6.13	4.73E-02
DNA replication (GO:0006260)	4.73	8.57E-04
DNA-templated DNA replication (GO:0006261)	4.64	3.44E-02
alpha-amino acid biosynthetic process (GO:1901607)	4.61	1.95E-07
tRNA metabolic process (GO:0006399)	4.29	2.26E-05
amino acid biosynthetic process (GO:0008652)	4.26	1.80E-07
proteinogenic amino acid biosynthetic process (GO:0170038)	3.95	7.86E-04
L-amino acid biosynthetic process (GO:0170034)	3.95	7.65E-04
amino acid metabolic process (GO:0006520)	3.86	4.08E-11
cytoplasmic translation (GO:0002181)	3.77	3.29E-02
ncRNA metabolic process (GO:0034660)	3.65	1.96E-05
translation (GO:0006412)	3.58	2.44E-04
alpha-amino acid metabolic process (GO:1901605)	3.54	2.84E-07
ribosome biogenesis (GO:0042254)	3.4	3.03E-03
ribonucleoprotein complex biogenesis (GO:0022613)	3.37	3.30E-03
RNA modification (GO:0009451)	3.27	1.16E-02

peptide biosynthetic process (GO:0043043)	3.25	7.66E-04
organic acid biosynthetic process (GO:0016053)	3.14	1.17E-05
carboxylic acid biosynthetic process (GO:0046394)	3.14	1.13E-05
proteinogenic amino acid metabolic process (GO:0170039)	3.11	5.83E-04
L-amino acid metabolic process (GO:0170033)	3.09	6.22E-04
ncRNA processing (GO:0034470)	2.95	1.78E-02
RNA processing (GO:0006396)	2.91	1.34E-02
peptide metabolic process (GO:0006518)	2.9	2.77E-03
organelle organization (GO:0006996)	2.78	9.79E-03
RNA metabolic process (GO:0016070)	2.75	2.21E-05
organonitrogen compound biosynthetic process (GO:1901566)	2.74	1.14E-11
amide biosynthetic process (GO:0043604)	2.7	4.32E-03
nucleic acid metabolic process (GO:0090304)	2.37	3.83E-07
small molecule biosynthetic process (GO:0044283)	2.29	1.03E-03
gene expression (GO:0010467)	2.24	1.26E-04
macromolecule biosynthetic process (GO:0009059)	2.21	3.63E-07
cellular biosynthetic process (GO:0044249)	2.2	6.47E-14
cellular nitrogen compound biosynthetic process (GO:0044271)	2.19	2.57E-05
organic substance biosynthetic process (GO:1901576)	2.16	4.42E-14
biosynthetic process (GO:0009058)	2.13	5.41E-14
cellular component biogenesis (GO:0044085)	2	9.76E-03

nucleobase-containing compound metabolic process (GO:0006139)	1.99	2.72E-06
cellular component organization or biogenesis (GO:0071840)	1.94	8.07E-04
macromolecule metabolic process (GO:0043170)	1.92	3.00E-08
cellular nitrogen compound metabolic process (GO:0034641)	1.9	1.67E-07
nitrogen compound metabolic process (GO:0006807)	1.88	5.76E-14
heterocycle metabolic process (GO:0046483)	1.84	1.05E-05
organic cyclic compound metabolic process (GO:1901360)	1.82	7.84E-06
cellular aromatic compound metabolic process (GO:0006725)	1.82	1.57E-05
cellular component organization (GO:0016043)	1.78	2.79E-02
organonitrogen compound metabolic process (GO:1901564)	1.77	1.15E-05
primary metabolic process (GO:0044238)	1.72	1.12E-11
cellular metabolic process (GO:0044237)	1.52	4.80E-08
organic substance metabolic process (GO:0071704)	1.51	4.40E-08
metabolic process (GO:0008152)	1.43	3.43E-07
cellular process (GO:0009987)	1.24	5.26E-05
biological_process (GO:0008150)	1.19	1.01E-05
Unclassified (UNCLASSIFIED)	0.36	9.62E-06

Table S10 Transcripts less abundant in ∆*tolC* NMM compared to ∆*tolC*

Gene ontology biological process	Fold Enrichment	p-value (FDR correction)

GMP biosynthetic process (GO:0006177)	3.87	1.66E-02
tripeptide import across plasma membrane (GO:0140207)	3.87	1.65E-02
'de novo' UMP biosynthetic process (GO:0044205)	3.87	1.64E-03
DNA-templated transcription termination (GO:0006353)	3.87	5.31E-03
CTP metabolic process (GO:0046036)	3.87	4.98E-02
dTDP-rhamnose biosynthetic process (GO:0019305)	3.87	4.96E-02
RNA 5'-end processing (GO:0000966)	3.87	4.94E-02
CTP biosynthetic process (GO:0006241)	3.87	4.92E-02
proton motive force-driven plasma membrane ATP synthesis (GO:0042777)	3.87	4.73E-04
protein insertion into membrane from inner side (GO:0032978)	3.87	4.69E-04
UDP-glucose metabolic process (GO:0006011)	3.87	4.90E-02
ncRNA 5'-end processing (GO:0034471)	3.87	4.88E-02
IMP salvage (GO:0032264)	3.87	4.86E-02
sister chromatid cohesion (GO:0007062)	3.87	4.85E-02
pyrimidine ribonucleoside triphosphate biosynthetic process (GO:0009209)	3.87	4.83E-02
dTDP-rhamnose metabolic process (GO:0046383)	3.87	4.81E-02
malonyl-CoA biosynthetic process (GO:2001295)	3.87	4.79E-02
malonyl-CoA metabolic process (GO:2001293)	3.87	4.78E-02

CDP-diacylglycerol metabolic process (GO:0046341)	3.87	4.76E-02
cytoplasmic translation (GO:0002181)	3.8	5.04E-30
ribosomal small subunit assembly (GO:0000028)	3.68	3.89E-09
protein-RNA complex organization (GO:0071826)	3.62	2.82E-20
protein-RNA complex assembly (GO:0022618)	3.62	2.68E-20
ribonucleoside triphosphate biosynthetic process (GO:0009201)	3.59	7.29E-06
pyrimidine ribonucleotide biosynthetic process (GO:0009220)	3.57	2.54E-05
ribosomal large subunit assembly (GO:0000027)	3.56	2.20E-10
ribosome assembly (GO:0042255)	3.55	2.08E-20
regulation of DNA-templated transcription elongation (GO:0032784)	3.52	2.82E-04
purine nucleoside triphosphate biosynthetic process (GO:0009145)	3.52	2.79E-04
purine ribonucleoside triphosphate biosynthetic process (GO:0009206)	3.52	2.77E-04
pyrimidine ribonucleoside monophosphate biosynthetic process (GO:0009174)	3.48	8.83E-04
UMP biosynthetic process (GO:0006222)	3.48	8.77E-04
'de novo' pyrimidine nucleobase biosynthetic process (GO:0006207)	3.44	2.82E-03
protein transport by the Sec complex (GO:0043952)	3.44	2.80E-03
ATP biosynthetic process (GO:0006754)	3.44	2.78E-03
electron transport coupled proton transport (GO:0015990)	3.44	2.76E-03

energy coupled proton transmembrane transport, against electrochemical gradient (GO:0015988)	3.44	2.74E-03
proton motive force-driven ATP synthesis (GO:0015986)	3.44	2.72E-03
ribosomal large subunit biogenesis (GO:0042273)	3.42	1.27E-09
thioester biosynthetic process (GO:0035384)	3.39	8.08E-03
enzyme-directed rRNA pseudouridine synthesis (GO:0000455)	3.39	8.04E-03
IMP biosynthetic process (GO:0006188)	3.39	1.20E-05
acyl-CoA biosynthetic process (GO:0071616)	3.39	7.99E-03
rRNA pseudouridine synthesis (GO:0031118)	3.39	7.95E-03
pyrimidine nucleoside monophosphate biosynthetic process (GO:0009130)	3.35	3.96E-05
DNA topological change (GO:0006265)	3.32	2.15E-02
purine ribonucleotide salvage (GO:0106380)	3.32	2.14E-02
maintenance of translational fidelity (GO:1990145)	3.32	2.13E-02
ribonucleoside monophosphate biosynthetic process (GO:0009156)	3.23	3.51E-11
'de novo' IMP biosynthetic process (GO:0006189)	3.23	1.12E-03
nucleoside monophosphate biosynthetic process (GO:0009124)	3.21	1.51E-12
purine ribonucleoside monophosphate biosynthetic process (GO:0009168)	3.2	6.32E-07

purine nucleoside monophosphate biosynthetic process (GO:0009127)	3.2	6.25E-07
IMP metabolic process (GO:0046040)	3.19	4.79E-05
purine nucleotide salvage (GO:0032261)	3.17	3.15E-03
translation (GO:0006412)	3.16	9.03E-36
ribosomal small subunit biogenesis (GO:0042274)	3.13	2.45E-07
ribonucleoprotein complex biogenesis (GO:0022613)	3.11	5.49E-29
ribosome biogenesis (GO:0042254)	3.1	1.66E-28
nucleobase biosynthetic process (GO:0046112)	3.1	1.81E-05
purine ribonucleoside monophosphate metabolic process (GO:0009167)	3.06	2.17E-06
purine nucleoside monophosphate metabolic process (GO:0009126)	3.06	2.15E-06
ribonucleotide biosynthetic process (GO:0009260)	3.06	1.77E-16
nucleoside triphosphate biosynthetic process (GO:0009142)	3.06	5.20E-05
pyrimidine nucleobase biosynthetic process (GO:0019856)	3.04	1.19E-03
intracellular transport (GO:0046907)	3.01	2.15E-02
intracellular protein transport (GO:0006886)	3.01	2.14E-02
intracellular protein transmembrane transport (GO:0065002)	3.01	2.13E-02
menaquinone biosynthetic process (GO:0009234)	3.01	2.12E-02
menaquinone metabolic process (GO:0009233)	3.01	2.11E-02
purine ribonucleotide biosynthetic process (GO:0009152)	2.98	7.23E-12

xenobiotic detoxification by transmembrane export across the cell outer membrane (GO:0140330)	2.98	3.19E-03
export across cell outer membrane (GO:0140317)	2.98	3.17E-03
ribose phosphate biosynthetic process (GO:0046390)	2.98	6.05E-16
ribonucleoside monophosphate metabolic process (GO:0009161)	2.98	1.28E-09
pyrimidine nucleoside monophosphate metabolic process (GO:0009129)	2.96	4.31E-04
nucleotide salvage (GO:0043173)	2.96	4.27E-04
peptide biosynthetic process (GO:0043043)	2.9	1.17E-30
pyrimidine ribonucleoside monophosphate metabolic process (GO:0009173)	2.9	8.44E-03
pseudouridine synthesis (GO:0001522)	2.9	8.40E-03
UMP metabolic process (GO:0046049)	2.9	8.35E-03
pyrimidine ribonucleotide metabolic process (GO:0009218)	2.9	1.17E-03
pyrimidine nucleotide biosynthetic process (GO:0006221)	2.86	5.12E-05
DNA replication initiation (GO:0006270)	2.82	2.01E-02
nucleoside monophosphate metabolic process (GO:0009123)	2.79	3.96E-10
tRNA aminoacylation for protein translation (GO:0006418)	2.79	4.84E-05
tRNA aminoacylation (GO:0043039)	2.79	4.80E-05
protein insertion into membrane (GO:0051205)	2.76	7.73E-03
purine nucleotide biosynthetic process (GO:0006164)	2.73	1.43E-11

DNA unwinding involved in DNA replication (GO:0006268)	2.71	4.80E-02
amide biosynthetic process (GO:0043604)	2.69	6.46E-31
amino acid activation (GO:0043038)	2.68	1.11E-04
peptide metabolic process (GO:0006518)	2.67	1.09E-26
non-membrane-bounded organelle assembly (GO:0140694)	2.65	1.15E-12
organelle assembly (GO:0070925)	2.65	1.12E-12
nucleoside bisphosphate biosynthetic process (GO:0033866)	2.58	1.54E-02
purine nucleoside bisphosphate biosynthetic process (GO:0034033)	2.58	1.53E-02
ribonucleoside bisphosphate biosynthetic process (GO:0034030)	2.58	1.53E-02
nucleotide biosynthetic process (GO:0009165)	2.58	7.85E-16
rRNA processing (GO:0006364)	2.58	3.30E-07
pyrimidine nucleotide metabolic process (GO:0006220)	2.58	2.46E-04
fatty acid biosynthetic process (GO:0006633)	2.58	7.02E-04
nucleoside phosphate biosynthetic process (GO:1901293)	2.58	7.58E-16
purine-containing compound biosynthetic process (GO:0072522)	2.56	3.51E-10
rRNA modification (GO:0000154)	2.54	6.69E-05
DNA-templated DNA replication (GO:0006261)	2.51	1.88E-05
thioester metabolic process (GO:0035383)	2.5	1.31E-02
establishment of protein localization to membrane (GO:0090150)	2.5	1.31E-02
acyl-CoA metabolic process (GO:0006637)	2.5	1.30E-02

translational elongation (GO:0006414)	2.49	3.23E-02
amino acid import across plasma membrane (GO:0089718)	2.49	4.72E-04
cellular component disassembly (GO:0022411)	2.49	3.22E-02
enterobacterial common antigen biosynthetic process (GO:0009246)	2.49	3.20E-02
enterobacterial common antigen metabolic process (GO:0046378)	2.49	3.19E-02
rRNA metabolic process (GO:0016072)	2.47	1.39E-06
rRNA base methylation (GO:0070475)	2.44	1.05E-02
tRNA modification (GO:0006400)	2.36	3.67E-07
ribonucleotide metabolic process (GO:0009259)	2.32	8.18E-11
negative regulation of termination of DNA-templated transcription (GO:0060567)	2.32	1.75E-02
transcription antitermination (GO:0031564)	2.32	1.74E-02
tRNA metabolic process (GO:0006399)	2.32	2.18E-11
negative regulation of protein-containing complex disassembly (GO:0043242)	2.32	1.74E-02
lipopolysaccharide core region metabolic process (GO:0046401)	2.32	5.47E-03
lipopolysaccharide core region biosynthetic process (GO:0009244)	2.32	5.43E-03
DNA replication (GO:0006260)	2.3	2.13E-06
oxidative phosphorylation (GO:0006119)	2.29	1.46E-02
RNA modification (GO:0009451)	2.27	7.92E-10
pyrimidine-containing compound biosynthetic process (GO:0072528)	2.27	3.31E-04

ncRNA metabolic process (GO:0034660)	2.25	1.48E-14
DNA conformation change (GO:0071103)	2.25	4.19E-03
ncRNA processing (GO:0034470)	2.24	5.59E-11
amide metabolic process (GO:0043603)	2.24	1.35E-21
negative regulation of translation (GO:0017148)	2.23	1.47E-02
nucleotide-sugar metabolic process (GO:0009225)	2.23	1.46E-02
purine ribonucleotide metabolic process (GO:0009150)	2.23	7.62E-08
RNA processing (GO:0006396)	2.23	8.11E-12
tRNA processing (GO:0008033)	2.23	2.83E-07
macromolecule methylation (GO:0043414)	2.21	9.19E-05
organelle organization (GO:0006996)	2.2	3.12E-13
rRNA methylation (GO:0031167)	2.19	3.21E-02
ribose phosphate metabolic process (GO:0019693)	2.19	5.80E-10
negative regulation of amide metabolic process (GO:0034249)	2.15	1.76E-02
RNA methylation (GO:0001510)	2.13	1.78E-03
positive regulation of gene expression (GO:0010628)	2.12	1.02E-02
regulation of protein-containing complex disassembly (GO:0043244)	2.12	1.01E-02
ribonucleoside bisphosphate metabolic process (GO:0033875)	2.08	3.07E-02
nucleoside bisphosphate metabolic process (GO:0033865)	2.08	3.05E-02
localization within membrane (GO:0051668)	2.08	3.04E-02
purine nucleoside bisphosphate metabolic process (GO:0034032)	2.08	3.03E-02

protein localization to membrane (GO:0072657)	2.08	3.02E-02
nucleoside triphosphate metabolic process (GO:0009141)	2.07	2.81E-03
regulation of termination of DNA-templated transcription (GO:0031554)	2.06	1.77E-02
ribonucleoside triphosphate metabolic process (GO:0009199)	2.05	1.64E-02
cellular nitrogen compound biosynthetic process (GO:0044271)	2.02	2.80E-36
negative regulation of cellular component organization (GO:0051129)	2.01	4.15E-02
organophosphate biosynthetic process (GO:0090407)	2	2.81E-13
oligosaccharide biosynthetic process (GO:0009312)	2	4.87E-02
negative regulation of protein metabolic process (GO:0051248)	2	3.39E-02
carbohydrate derivative biosynthetic process (GO:1901137)	2	1.78E-17
gene expression (GO:0010467)	1.98	3.81E-28
purine nucleotide metabolic process (GO:0006163)	1.97	3.53E-07
regulation of translation (GO:0006417)	1.97	8.09E-04
chromosome organization (GO:0051276)	1.94	4.52E-03
purine nucleoside triphosphate metabolic process (GO:0009144)	1.94	3.07E-02
regulation of amide metabolic process (GO:0034248)	1.94	1.56E-03
L-alpha-amino acid transmembrane transport (GO:1902475)	1.94	1.53E-02
methylation (GO:0032259)	1.94	8.10E-04

nucleotide metabolic process (GO:0009117)	1.92	9.65E-10
organonitrogen compound biosynthetic process (GO:1901566)	1.9	1.01E-32
nucleoside phosphate metabolic process (GO:0006753)	1.9	1.59E-09
aerobic respiration (GO:0009060)	1.87	3.10E-03
macromolecule modification (GO:0043412)	1.87	1.02E-07
negative regulation of gene expression (GO:0010629)	1.86	1.01E-02
glycosaminoglycan biosynthetic process (GO:0006024)	1.86	1.37E-02
aminoglycan biosynthetic process (GO:0006023)	1.86	1.37E-02
peptidoglycan biosynthetic process (GO:0009252)	1.86	1.36E-02
macromolecule biosynthetic process (GO:0009059)	1.85	3.34E-34
post-transcriptional regulation of gene expression (GO:0010608)	1.85	2.94E-03
xenobiotic transport (GO:0042908)	1.84	3.65E-02
nucleobase-containing compound biosynthetic process (GO:0034654)	1.83	2.96E-12
response to antibiotic (GO:0046677)	1.83	4.80E-05
peptidoglycan-based cell wall biogenesis (GO:0009273)	1.83	1.22E-02
protein-containing complex organization (GO:0043933)	1.82	4.70E-08
cell wall macromolecule biosynthetic process (GO:0044038)	1.82	1.55E-02
proton transmembrane transport (GO:1902600)	1.81	4.69E-03
lipid biosynthetic process (GO:0008610)	1.8	2.57E-06
response to radiation (GO:0009314)	1.8	2.67E-04
cell wall biogenesis (GO:0042546)	1.79	1.93E-02

L-amino acid transport (GO:0015807)	1.78	2.34E-02
protein-containing complex assembly (GO:0065003)	1.74	3.14E-06
RNA metabolic process (GO:0016070)	1.74	2.20E-10
phospholipid biosynthetic process (GO:0008654)	1.74	2.64E-02
amino acid transmembrane transport (GO:0003333)	1.73	7.37E-03
cellular biosynthetic process (GO:0044249)	1.71	1.26E-47
phospholipid metabolic process (GO:0006644)	1.71	2.22E-02
pyrimidine-containing compound metabolic process (GO:0072527)	1.7	2.09E-02
protein metabolic process (GO:0019538)	1.7	1.09E-12
organic substance biosynthetic process (GO:1901576)	1.7	1.90E-47
liposaccharide metabolic process (GO:1903509)	1.68	6.70E-03
biosynthetic process (GO:0009058)	1.68	4.08E-46
nucleobase-containing small molecule metabolic process (GO:0055086)	1.68	3.35E-08
inorganic cation transmembrane transport (GO:0098662)	1.67	3.78E-04
purine-containing compound metabolic process (GO:0072521)	1.65	3.78E-04
aromatic compound biosynthetic process (GO:0019438)	1.65	2.16E-10
monoatomic cation transmembrane transport (GO:0098655)	1.65	4.85E-04
amino acid transport (GO:0006865)	1.64	6.65E-03
cellular component biogenesis (GO:0044085)	1.63	7.51E-11

peptidoglycan metabolic process (GO:0000270)	1.62	2.34E-02
glycosaminoglycan metabolic process (GO:0030203)	1.62	2.33E-02
regulation of protein metabolic process (GO:0051246)	1.62	3.34E-02
heterocycle biosynthetic process (GO:0018130)	1.62	4.01E-10
lipopolysaccharide biosynthetic process (GO:0009103)	1.61	4.48E-02
organic cyclic compound biosynthetic process (GO:1901362)	1.61	1.85E-10
carbohydrate derivative metabolic process (GO:1901135)	1.61	2.53E-11
cellular component organization or biogenesis (GO:0071840)	1.6	4.57E-15
cellular nitrogen compound metabolic process (GO:0034641)	1.6	5.63E-31
organophosphate metabolic process (GO:0019637)	1.6	9.03E-08
inorganic ion transmembrane transport (GO:0098660)	1.59	8.82E-04
cell wall organization or biogenesis (GO:0071554)	1.59	1.75E-02
aminoglycan metabolic process (GO:0006022)	1.59	2.80E-02
polysaccharide biosynthetic process (GO:0000271)	1.54	1.51E-02
monoatomic ion transmembrane transport (GO:0034220)	1.51	2.95E-03
nucleobase-containing compound metabolic process (GO:0006139)	1.5	1.64E-15
cellular component organization (GO:0016043)	1.48	1.33E-08
carbohydrate biosynthetic process (GO:0016051)	1.47	2.23E-02

macromolecule metabolic process (GO:0043170)	1.47	2.78E-20
lipid metabolic process (GO:0006629)	1.47	2.71E-03
monoatomic cation transport (GO:0006812)	1.47	5.65E-03
polysaccharide metabolic process (GO:0005976)	1.46	4.04E-02
cellular lipid metabolic process (GO:0044255)	1.44	4.62E-02
heterocycle metabolic process (GO:0046483)	1.43	1.98E-14
nucleic acid metabolic process (GO:0090304)	1.4	1.12E-06
nitrogen compound transport (GO:0071705)	1.4	2.20E-03
cellular component assembly (GO:0022607)	1.4	2.94E-03
cellular aromatic compound metabolic process (GO:0006725)	1.4	2.03E-12
organic cyclic compound metabolic process (GO:1901360)	1.39	1.35E-12
organonitrogen compound metabolic process (GO:1901564)	1.38	4.48E-13
phosphate-containing compound metabolic process (GO:0006796)	1.37	1.10E-04
phosphorus metabolic process (GO:0006793)	1.36	8.57E-05
small molecule biosynthetic process (GO:0044283)	1.36	3.08E-03
monoatomic ion transport (GO:0006811)	1.36	2.16E-02
nitrogen compound metabolic process (GO:0006807)	1.35	3.40E-19
response to abiotic stimulus (GO:0009628)	1.33	1.89E-02

primary metabolic process (GO:0044238)	1.29	8.57E-17
cellular metabolic process (GO:0044237)	1.26	9.98E-18
organic substance metabolic process (GO:0071704)	1.22	4.24E-13
metabolic process (GO:0008152)	1.2	1.56E-12
cellular process (GO:0009987)	1.17	1.83E-20
biological_process (GO:0008150)	1.11	3.57E-14
regulation of RNA metabolic process (GO:0051252)	0.74	2.23E-02
regulation of RNA biosynthetic process (GO:2001141)	0.73	1.76E-02
regulation of DNA-templated transcription (GO:0006355)	0.73	1.75E-02
organonitrogen compound catabolic process (GO:1901565)	0.67	2.32E-02
Unclassified (UNCLASSIFIED)	0.63	3.68E-14
organic substance catabolic process (GO:1901575)	0.63	1.44E-06
cellular catabolic process (GO:0044248)	0.63	1.23E-04
response to external stimulus (GO:0009605)	0.62	1.68E-02
catabolic process (GO:0009056)	0.61	1.70E-07
carboxylic acid catabolic process (GO:0046395)	0.58	3.59E-03
organic acid catabolic process (GO:0016054)	0.57	1.60E-03
cell communication (GO:0007154)	0.55	3.76E-03
small molecule catabolic process (GO:0044282)	0.55	5.17E-06
signaling (GO:0023052)	0.5	3.02E-02
carbohydrate transmembrane transport (GO:0034219)	0.49	1.36E-02
carbohydrate transport (GO:0008643)	0.48	3.91E-03

intracellular signal transduction (GO:0035556)	0.46	4.01E-02
phosphorelay signal transduction system (GO:0000160)	0.42	3.22E-02
DNA transposition (GO:0006313)	0.37	4.71E-02
transposition (GO:0032196)	0.35	1.08E-02
cellular response to xenobiotic stimulus (GO:0071466)	0.12	2.14E-02
xenobiotic metabolic process (GO:0006805)	0.12	2.13E-02
benzene-containing compound metabolic process (GO:0042537)	0.12	1.54E-02
primary alcohol catabolic process (GO:0034310)	< 0.01	1.93E-02
cellular biogenic amine catabolic process (GO:0042402)	< 0.01	3.75E-03
xenobiotic catabolic process (GO:0042178)	< 0.01	8.90E-03
amine catabolic process (GO:0009310)	< 0.01	3.72E-03

Table S11 Transcripts more abundant in Δ*tolC* NMM compared to Δ*tolC*

Gene ontology biological process	Fold Enrichment	p-value (FDR correction)
xanthine metabolic process (GO:0046110)	> 100	2.03E-02
glutaminyl-tRNAGln biosynthesis via transamidation (GO:0070681)	> 100	4.77E-04
deoxyadenosine metabolic process (GO:0046090)	> 100	4.11E-02
deoxyadenosine catabolic process (GO:0006157)	> 100	3.97E-02
deoxyribonucleoside catabolic process (GO:0046121)	99	3.43E-03
deoxyribonucleoside metabolic process (GO:0009120)	54	1.70E-02

serine family amino acid biosynthetic process (GO:0009070)	33	4.75E-02
nucleoside catabolic process (GO:0009164)	31.68	7.79E-03
glycosyl compound catabolic process (GO:1901658)	29.12	1.61E-03
nucleobase-containing small molecule catabolic process (GO:0034656)	28.29	1.16E-02
serine family amino acid metabolic process (GO:0009069)	23.29	1.93E-02
carbohydrate catabolic process (GO:0016052)	14.4	4.38E-04
glycosyl compound metabolic process (GO:1901657)	11.65	4.16E-02
purine ribonucleotide catabolic process (GO:0009154)	10.76	4.93E-02
nucleotide catabolic process (GO:0009166)	9.5	2.74E-02
nucleoside phosphate catabolic process (GO:1901292)	8.87	3.72E-02
amino acid metabolic process (GO:0006520)	7.48	6.57E-04
small molecule catabolic process (GO:0044282)	7.27	2.10E-04
carbohydrate derivative catabolic process (GO:1901136)	7.07	1.73E-02
nucleobase-containing compound catabolic process (GO:0034655)	5.3	3.30E-02
carbohydrate metabolic process (GO:0005975)	4.44	4.44E-02
cellular catabolic process (GO:0044248)	3.5	1.58E-03
amide metabolic process (GO:0043603)	3.35	4.04E-02
organonitrogen compound catabolic process (GO:1901565)	3.34	3.76E-03

organic substance catabolic process (GO:1901575)	3.27	3.91E-04
catabolic process (GO:0009056)	2.86	6.80E-04
small molecule metabolic process (GO:0044281)	2.66	1.73E-02
organonitrogen compound metabolic process (GO:1901564)	1.82	1.94E-02
cellular metabolic process (GO:0044237)	1.73	2.88E-03
organic substance metabolic process (GO:0071704)	1.62	3.82E-03
metabolic process (GO:0008152)	1.61	2.26E-03
primary metabolic process (GO:0044238)	1.6	1.86E-02

Table S12 Transcripts less abundant in ∆*tolC tufA::kan* NMM compared to ∆*tolC* NMM

Gene ontology biological process	Fold Enrichment	p-value (FDR correction)
galactarate metabolic process (GO:0019580)	4.11	6.80E-03
aldaric acid catabolic process (GO:0019579)	4.11	2.19E-02
aldaric acid metabolic process (GO:0019577)	4.11	2.39E-03
hydrogen sulfide biosynthetic process (GO:0070814)	4.11	6.72E-03
hydrogen sulfide metabolic process (GO:0070813)	4.11	6.65E-03
glucarate metabolic process (GO:0019392)	4.11	6.58E-03
D-glucarate metabolic process (GO:0042836)	4.11	6.51E-03
sulfate assimilation (GO:0000103)	3.65	3.58E-03
lysine catabolic process (GO:0006554)	3.52	2.69E-02

uracil catabolic process (GO:0006212)	3.28	9.50E-03
macrolide metabolic process (GO:0033067)	3.19	2.70E-02
enterobactin metabolic process (GO:0009238)	3.19	2.68E-02
polyketide metabolic process (GO:0030638)	3.19	2.66E-02
phenol-containing compound metabolic process (GO:0018958)	3.08	9.01E-03
pyrimidine nucleobase catabolic process (GO:0006208)	3.08	8.92E-03
catechol-containing compound metabolic process (GO:0009712)	2.99	2.42E-02
uracil metabolic process (GO:0019860)	2.99	2.40E-02
antibiotic metabolic process (GO:0016999)	2.74	4.95E-02
pyrimidine-containing compound catabolic process (GO:0072529)	2.35	2.95E-02
cellular response to starvation (GO:0009267)	2.15	3.53E-03
cellular response to nutrient levels (GO:0031669)	2.1	5.24E-03
response to starvation (GO:0042594)	2.05	2.68E-03
organic hydroxy compound catabolic process (GO:1901616)	1.97	7.04E-03
alcohol catabolic process (GO:0046164)	1.96	1.92E-02
response to nutrient levels (GO:0031667)	1.82	2.89E-03
cellular response to external stimulus (GO:0071496)	1.79	5.85E-03
cellular response to extracellular stimulus (GO:0031668)	1.79	5.78E-03
response to extracellular stimulus (GO:0009991)	1.69	2.62E-03
response to oxidative stress (GO:0006979)	1.65	1.09E-02

cellular response to stress (GO:0033554)	1.48	8.34E-06
DNA damage response (GO:0006974)	1.42	2.91E-03
response to stress (GO:0006950)	1.42	8.11E-07
cellular response to stimulus (GO:0051716)	1.36	7.08E-05
small molecule catabolic process (GO:0044282)	1.35	8.25E-03
catabolic process (GO:0009056)	1.3	2.25E-03
organic substance catabolic process (GO:1901575)	1.29	3.51E-03
cellular catabolic process (GO:0044248)	1.28	4.49E-02
Unclassified (UNCLASSIFIED)	1.24	1.53E-04
response to stimulus (GO:0050896)	1.23	2.27E-04
biological_process (GO:0008150)	0.93	1.48E-04
cellular process (GO:0009987)	0.88	2.39E-07
metabolic process (GO:0008152)	0.88	6.02E-04
organic substance metabolic process (GO:0071704)	0.86	3.84E-04
cellular metabolic process (GO:0044237)	0.85	8.75E-05
primary metabolic process (GO:0044238)	0.85	3.97E-04
organonitrogen compound metabolic process (GO:1901564)	0.82	6.94E-03
localization (GO:0051179)	0.78	6.76E-03
transport (GO:0006810)	0.78	6.48E-03
nitrogen compound metabolic process (GO:0006807)	0.78	6.74E-07
establishment of localization (GO:0051234)	0.77	4.33E-03
macromolecule metabolic process (GO:0043170)	0.75	3.39E-05
transmembrane transport (GO:0055085)	0.75	3.33E-03

cellular aromatic compound metabolic process (GO:0006725)	0.73	2.29E-05
organic cyclic compound metabolic process (GO:1901360)	0.72	7.70E-06
carbohydrate derivative metabolic process (GO:1901135)	0.71	1.14E-02
heterocycle metabolic process (GO:0046483)	0.7	1.18E-06
organic substance transport (GO:0071702)	0.69	1.32E-03
nucleobase-containing compound metabolic process (GO:0006139)	0.68	8.03E-06
biosynthetic process (GO:0009058)	0.68	1.29E-09
organic substance biosynthetic process (GO:1901576)	0.67	2.11E-10
macromolecule biosynthetic process (GO:0009059)	0.66	2.32E-05
nucleic acid metabolic process (GO:0090304)	0.66	1.53E-04
gene expression (GO:0010467)	0.65	1.37E-03
heterocycle biosynthetic process (GO:0018130)	0.64	2.32E-03
cellular biosynthetic process (GO:0044249)	0.64	9.62E-12
cellular nitrogen compound metabolic process (GO:0034641)	0.64	9.29E-11
RNA metabolic process (GO:0016070)	0.62	6.48E-03
organic cyclic compound biosynthetic process (GO:1901362)	0.62	4.37E-04
cellular component biogenesis (GO:0044085)	0.61	3.18E-04
aromatic compound biosynthetic process (GO:0019438)	0.6	6.50E-04
cellular component organization (GO:0016043)	0.6	1.35E-05
amide metabolic process (GO:0043603)	0.6	1.13E-02

nucleobase-containing compound biosynthetic process (GO:0034654)	0.59	3.93E-03
cellular component organization or biogenesis (GO:0071840)	0.58	3.32E-07
carbohydrate derivative biosynthetic process (GO:1901137)	0.53	3.20E-04
import into cell (GO:0098657)	0.52	1.32E-02
small molecule biosynthetic process (GO:0044283)	0.52	2.24E-05
cellular nitrogen compound biosynthetic process (GO:0044271)	0.51	6.66E-09
alpha-amino acid biosynthetic process (GO:1901607)	0.51	2.78E-02
cellular localization (GO:0051641)	0.5	1.59E-02
monoatomic cation transmembrane transport (GO:0098655)	0.49	1.23E-02
organic acid biosynthetic process (GO:0016053)	0.48	3.89E-04
carboxylic acid biosynthetic process (GO:0046394)	0.48	3.80E-04
inorganic cation transmembrane transport (GO:0098662)	0.47	7.84E-03
peptide biosynthetic process (GO:0043043)	0.47	6.57E-03
DNA recombination (GO:0006310)	0.46	8.27E-03
organonitrogen compound biosynthetic process (GO:1901566)	0.45	9.92E-13
carbohydrate transport (GO:0008643)	0.45	5.57E-03
import across plasma membrane (GO:0098739)	0.44	5.76E-03
cell wall organization or biogenesis (GO:0071554)	0.43	2.38E-02
amide biosynthetic process (GO:0043604)	0.42	2.74E-04
cell wall organization (GO:0071555)	0.42	4.59E-02
amino acid biosynthetic process (GO:0008652)	0.41	8.74E-04

peptidoglycan metabolic process (GO:0000270)	0.41	3.42E-02
glycosaminoglycan metabolic process (GO:0030203)	0.41	3.39E-02
tRNA processing (GO:0008033)	0.39	4.76E-02
RNA processing (GO:0006396)	0.38	2.28E-03
ncRNA processing (GO:0034470)	0.38	2.80E-03
organelle organization (GO:0006996)	0.35	2.24E-04
macromolecule localization (GO:0033036)	0.34	9.00E-05
protein transport (GO:0015031)	0.34	7.84E-03
cellular macromolecule localization (GO:0070727)	0.34	3.70E-03
protein localization (GO:0008104)	0.34	3.65E-03
nucleotide biosynthetic process (GO:0009165)	0.33	2.24E-03
nucleoside phosphate biosynthetic process (GO:1901293)	0.33	2.20E-03
tRNA metabolic process (GO:0006399)	0.33	1.62E-03
proton transmembrane transport (GO:1902600)	0.32	2.54E-02
ncRNA metabolic process (GO:0034660)	0.32	4.97E-05
RNA modification (GO:0009451)	0.31	2.17E-03
establishment of protein localization (GO:0045184)	0.31	2.17E-03
translation (GO:0006412)	0.27	5.22E-05
cell projection organization (GO:0030030)	0.26	2.29E-03
rRNA metabolic process (GO:0016072)	0.26	4.25E-02
purine-containing compound biosynthetic process (GO:0072522)	0.25	6.86E-03
ribose phosphate biosynthetic process (GO:0046390)	0.25	6.78E-03
phosphoenolpyruvate-dependent sugar phosphotransferase system (GO:0009401)	0.21	4.22E-03

purine nucleotide biosynthetic process (GO:0006164)	0.2	4.34E-03
nucleoside monophosphate metabolic process (GO:0009123)	0.16	6.54E-03
ribosome biogenesis (GO:0042254)	0.16	9.13E-06
ribonucleoprotein complex biogenesis (GO:0022613)	0.16	7.40E-06
ribonucleotide biosynthetic process (GO:0009260)	0.13	5.68E-04
membrane organization (GO:0061024)	0.12	2.68E-02
ribonucleoside monophosphate biosynthetic process (GO:0009156)	0.11	1.93E-02
non-membrane-bounded organelle assembly (GO:0140694)	0.11	7.24E-05
organelle assembly (GO:0070925)	0.11	6.96E-05
ribonucleoside monophosphate metabolic process (GO:0009161)	0.11	1.30E-02
nucleoside monophosphate biosynthetic process (GO:0009124)	0.1	9.03E-03
purine ribonucleotide biosynthetic process (GO:0009152)	0.09	2.46E-03
cytoplasmic translation (GO:0002181)	< 0.01	2.31E-05
defense response to other organism (GO:0098542)	< 0.01	2.46E-02
defense response (GO:0006952)	< 0.01	2.44E-02
protein-RNA complex organization (GO:0071826)	< 0.01	3.83E-04
protein transmembrane transport (GO:0071806)	< 0.01	1.30E-03
ribosomal large subunit biogenesis (GO:0042273)	< 0.01	2.41E-02
ribosome assembly (GO:0042255)	< 0.01	1.68E-04
protein-RNA complex assembly (GO:0022618)	< 0.01	3.73E-04
ribosomal large subunit assembly (GO:0000027)	< 0.01	3.54E-02

Table S13 Transcripts more abundant in Δ*tolC tufA::kan* NMM compared to Δ*tolC* NMM

Gene ontology biological process	Fold Enrichment	p-value (FDR correction)
purine nucleoside triphosphate biosynthetic process (GO:0009145)	4.06	1.03E-05
thioester biosynthetic process (GO:0035384)	4.06	4.27E-04
maltodextrin transmembrane transport (GO:0042956)	4.06	1.55E-02
dextrin transport (GO:0042955)	4.06	1.54E-02
dTDP-rhamnose biosynthetic process (GO:0019305)	4.06	4.91E-02
GMP biosynthetic process (GO:0006177)	4.06	1.53E-02
regulation of membrane invagination (GO:1905153)	4.06	1.52E-02
proton motive force-driven plasma membrane ATP synthesis (GO:0042777)	4.06	4.23E-04
acyl-CoA biosynthetic process (GO:0071616)	4.06	4.18E-04
acetyl-CoA biosynthetic process (GO:0006085)	4.06	4.89E-02
tripeptide import across plasma membrane (GO:0140207)	4.06	1.51E-02
IMP salvage (GO:0032264)	4.06	4.87E-02
purine ribonucleoside triphosphate biosynthetic process (GO:0009206)	4.06	1.01E-05
dTDP-rhamnose metabolic process (GO:0046383)	4.06	4.84E-02
malonyl-CoA biosynthetic process (GO:2001295)	4.06	4.82E-02
malonyl-CoA metabolic process (GO:2001293)	4.06	4.80E-02
maltose transport (GO:0015768)	4.06	4.84E-03
ATP biosynthetic process (GO:0006754)	4.06	1.25E-04

proton motive force-driven ATP synthesis (GO:0015986)	4.06	1.23E-04
cytoplasmic translation (GO:0002181)	3.99	1.81E-30
ribonucleoside triphosphate biosynthetic process (GO:0009201)	3.77	7.17E-06
electron transport coupled proton transport (GO:0015990)	3.61	2.45E-03
energy coupled proton transmembrane transport, against electrochemical gradient (GO:0015988)	3.61	2.43E-03
leucine biosynthetic process (GO:0009098)	3.55	7.20E-03
protein insertion into membrane from inner side (GO:0032978)	3.55	7.15E-03
leucine metabolic process (GO:0006551)	3.55	7.10E-03
galactose transmembrane transport (GO:0015757)	3.48	1.99E-02
purine ribonucleotide salvage (GO:0106380)	3.48	1.98E-02
maintenance of translational fidelity (GO:1990145)	3.48	1.97E-02
ribosomal small subunit assembly (GO:0000028)	3.45	1.41E-06
protein-RNA complex organization (GO:0071826)	3.44	1.35E-15
protein-RNA complex assembly (GO:0022618)	3.44	1.29E-15
ribosomal large subunit assembly (GO:0000027)	3.41	4.93E-08
ribosome assembly (GO:0042255)	3.4	5.89E-16
regulation of DNA-templated transcription elongation (GO:0032784)	3.32	2.64E-03
purine nucleotide salvage (GO:0032261)	3.32	2.62E-03
ribosomal large subunit biogenesis (GO:0042273)	3.28	1.87E-07
protein transport by the Sec complex (GO:0043952)	3.16	1.97E-02

xenobiotic detoxification by transmembrane export across the cell outer membrane (GO:0140330)	3.12	2.65E-03
export across cell outer membrane (GO:0140317)	3.12	2.63E-03
translation (GO:0006412)	3.11	1.87E-30
nucleotide salvage (GO:0043173)	3.1	3.38E-04
translational termination (GO:0006415)	3.04	4.91E-02
plasmid maintenance (GO:0006276)	3.04	4.89E-02
protein-containing complex disassembly (GO:0032984)	3.04	4.87E-02
nucleoside triphosphate biosynthetic process (GO:0009142)	2.99	3.13E-04
ribosomal small subunit biogenesis (GO:0042274)	2.97	1.28E-05
non-membrane-bounded organelle assembly (GO:0140694)	2.89	4.93E-15
organelle assembly (GO:0070925)	2.89	4.71E-15
thioester metabolic process (GO:0035383)	2.87	2.22E-03
acyl-CoA metabolic process (GO:0006637)	2.87	2.20E-03
peptide biosynthetic process (GO:0043043)	2.83	8.55E-26
ribonucleoprotein complex biogenesis (GO:0022613)	2.83	1.03E-19
ribosome biogenesis (GO:0042254)	2.81	2.81E-19
tRNA aminoacylation for protein translation (GO:0006418)	2.76	2.00E-04
branched-chain amino acid biosynthetic process (GO:0009082)	2.76	1.97E-04
branched-chain amino acid metabolic process (GO:0009081)	2.76	1.95E-04
tRNA aminoacylation (GO:0043039)	2.76	1.93E-04
nucleoside bisphosphate biosynthetic process (GO:0033866)	2.71	1.27E-02

purine nucleoside bisphosphate biosynthetic process (GO:0034033)	2.71	1.26E-02
ribonucleoside bisphosphate biosynthetic process (GO:0034030)	2.71	1.25E-02
chromosome segregation (GO:0007059)	2.71	1.24E-02
amino acid activation (GO:0043038)	2.65	4.01E-04
purine ribonucleotide biosynthetic process (GO:0009152)	2.62	3.15E-07
protein insertion into membrane (GO:0051205)	2.61	2.79E-02
enterobacterial common antigen biosynthetic process (GO:0009246)	2.61	2.77E-02
enterobacterial common antigen metabolic process (GO:0046378)	2.61	2.76E-02
amide biosynthetic process (GO:0043604)	2.6	1.37E-25
ribonucleotide biosynthetic process (GO:0009260)	2.55	1.25E-08
peptide metabolic process (GO:0006518)	2.55	3.76E-21
ribose phosphate biosynthetic process (GO:0046390)	2.5	1.82E-08
oligosaccharide transport (GO:0015772)	2.48	1.76E-02
amino acid import across plasma membrane (GO:0089718)	2.46	1.41E-03
purine nucleotide biosynthetic process (GO:0006164)	2.46	1.45E-07
negative regulation of termination of DNA-templated transcription (GO:0060567)	2.44	1.40E-02
pyrimidine nucleoside monophosphate biosynthetic process (GO:0009130)	2.44	4.81E-02
disaccharide transport (GO:0015766)	2.44	4.79E-02
isoleucine biosynthetic process (GO:0009097)	2.44	4.77E-02
transcription antitermination (GO:0031564)	2.44	1.39E-02
negative regulation of protein-containing complex disassembly (GO:0043242)	2.44	1.38E-02

purine-containing compound salvage (GO:0043101)	2.44	4.75E-02
isoleucine metabolic process (GO:0006549)	2.44	4.72E-02
oxidative phosphorylation (GO:0006119)	2.4	1.09E-02
organelle organization (GO:0006996)	2.34	5.43E-15
purine-containing compound biosynthetic process (GO:0072522)	2.31	1.98E-06
nucleoside monophosphate biosynthetic process (GO:0009124)	2.28	7.13E-04
ribonucleoside monophosphate biosynthetic process (GO:0009156)	2.26	2.57E-03
positive regulation of gene expression (GO:0010628)	2.23	9.01E-03
regulation of protein-containing complex disassembly (GO:0043244)	2.23	8.95E-03
nucleotide biosynthetic process (GO:0009165)	2.21	1.16E-08
nucleoside phosphate biosynthetic process (GO:1901293)	2.21	1.13E-08
fatty acid biosynthetic process (GO:0006633)	2.2	3.94E-02
taxis (GO:0042330)	2.19	3.22E-03
chemotaxis (GO:0006935)	2.19	3.20E-03
DNA-templated DNA replication (GO:0006261)	2.19	3.17E-03
locomotion (GO:0040011)	2.19	3.15E-03
ribonucleoside bisphosphate metabolic process (GO:0033875)	2.19	2.84E-02
nucleoside bisphosphate metabolic process (GO:0033865)	2.19	2.83E-02
negative regulation of translation (GO:0017148)	2.19	2.81E-02
purine nucleoside bisphosphate metabolic process (GO:0034032)	2.19	2.80E-02
regulation of termination of DNA-templated transcription (GO:0031554)	2.17	1.49E-02

amide metabolic process (GO:0043603)	2.13	4.37E-17
tRNA metabolic process (GO:0006399)	2.11	1.88E-07
negative regulation of amide metabolic process (GO:0034249)	2.11	3.51E-02
ribonucleoside monophosphate metabolic process (GO:0009161)	2.08	9.94E-03
rRNA processing (GO:0006364)	2.08	3.43E-03
tRNA modification (GO:0006400)	2.06	4.03E-04
DNA replication (GO:0006260)	2.06	3.99E-04
bacterial-type flagellum organization (GO:0044781)	2.03	2.70E-02
ribonucleoside triphosphate metabolic process (GO:0009199)	2.03	2.82E-02
purine nucleoside triphosphate metabolic process (GO:0009144)	2.03	2.81E-02
nucleoside monophosphate metabolic process (GO:0009123)	2.03	2.51E-03
rRNA modification (GO:0000154)	2.03	2.69E-02
tricarboxylic acid cycle (GO:0006099)	2.03	3.48E-02
purine ribonucleoside triphosphate metabolic process (GO:0009205)	2.03	3.47E-02
dicarboxylic acid biosynthetic process (GO:0043650)	2.03	2.09E-02
rRNA metabolic process (GO:0016072)	1.99	5.88E-03
ncRNA metabolic process (GO:0034660)	1.99	1.84E-08
nucleoside triphosphate metabolic process (GO:0009141)	1.98	1.05E-02
purine ribonucleotide metabolic process (GO:0009150)	1.98	1.33E-04
neutral amino acid transport (GO:0015804)	1.98	2.55E-02
ribonucleotide metabolic process (GO:0009259)	1.97	1.89E-05
aerobic respiration (GO:0009060)	1.96	1.69E-03
tRNA processing (GO:0008033)	1.95	4.21E-04
RNA modification (GO:0009451)	1.94	4.53E-05

L-alpha-amino acid transmembrane transport (GO:1902475)	1.93	2.62E-02
RNA processing (GO:0006396)	1.93	3.25E-06
ribose phosphate metabolic process (GO:0019693)	1.92	1.60E-05
proton transmembrane transport (GO:1902600)	1.9	2.55E-03
ncRNA processing (GO:0034470)	1.9	1.90E-05
purine nucleotide metabolic process (GO:0006163)	1.88	2.40E-05
protein transport (GO:0015031)	1.88	4.68E-04
protein transmembrane transport (GO:0071806)	1.88	3.93E-02
archaeal or bacterial-type flagellum-dependent cell motility (GO:0097588)	1.85	1.03E-02
bacterial-type flagellum-dependent cell motility (GO:0071973)	1.85	1.02E-02
cilium or flagellum-dependent cell motility (GO:0001539)	1.85	1.02E-02
organonitrogen compound biosynthetic process (GO:1901566)	1.85	7.26E-27
cell motility (GO:0048870)	1.84	7.19E-03
gene expression (GO:0010467)	1.83	1.43E-18
macromolecule methylation (GO:0043414)	1.82	3.09E-02
cellular nitrogen compound biosynthetic process (GO:0044271)	1.81	1.16E-21
negative regulation of gene expression (GO:0010629)	1.8	2.78E-02
establishment of protein localization (GO:0045184)	1.79	1.20E-03
L-amino acid transport (GO:0015807)	1.79	3.60E-02
cellular macromolecule localization (GO:0070727)	1.78	1.03E-03
protein localization (GO:0008104)	1.78	1.02E-03
amino acid transmembrane transport (GO:0003333)	1.76	1.03E-02

macromolecule localization (GO:0033036)	1.73	6.29E-05
nucleotide metabolic process (GO:0009117)	1.72	1.94E-05
regulation of translation (GO:0006417)	1.72	4.46E-02
nucleoside phosphate metabolic process (GO:0006753)	1.71	1.91E-05
regulation of amide metabolic process (GO:0034248)	1.69	4.82E-02
response to antibiotic (GO:0046677)	1.69	3.15E-03
protein-containing complex organization (GO:0043933)	1.68	5.01E-05
macromolecule biosynthetic process (GO:0009059)	1.65	6.16E-19
regulation of cellular component organization (GO:0051128)	1.65	4.09E-02
amino acid transport (GO:0006865)	1.63	1.32E-02
response to radiation (GO:0009314)	1.63	1.31E-02
inorganic cation transmembrane transport (GO:0098662)	1.62	2.61E-03
protein-containing complex assembly (GO:0065003)	1.62	4.25E-04
respiratory electron transport chain (GO:0022904)	1.61	4.32E-02
purine-containing compound metabolic process (GO:0072521)	1.61	2.56E-03
protein metabolic process (GO:0019538)	1.61	8.17E-09
macromolecule modification (GO:0043412)	1.61	1.16E-03
monoatomic cation transmembrane transport (GO:0098655)	1.61	2.51E-03
organophosphate biosynthetic process (GO:0090407)	1.6	1.29E-04
carbohydrate derivative biosynthetic process (GO:1901137)	1.6	4.75E-06
nucleobase-containing compound biosynthetic process (GO:0034654)	1.6	7.21E-06

cellular localization (GO:0051641)	1.58	5.88E-03
cellular biosynthetic process (GO:0044249)	1.56	7.12E-28
import across plasma membrane (GO:0098739)	1.56	1.09E-02
inorganic ion transmembrane transport (GO:0098660)	1.56	3.48E-03
RNA metabolic process (GO:0016070)	1.55	2.10E-05
nitrogen compound transport (GO:0071705)	1.54	1.94E-05
organic substance biosynthetic process (GO:1901576)	1.54	8.90E-27
amino acid biosynthetic process (GO:0008652)	1.53	9.70E-03
organic acid biosynthetic process (GO:0016053)	1.52	8.50E-04
carboxylic acid biosynthetic process (GO:0046394)	1.52	8.42E-04
biosynthetic process (GO:0009058)	1.52	1.41E-25
cellular component organization or biogenesis (GO:0071840)	1.51	4.28E-10
monoatomic ion transmembrane transport (GO:0034220)	1.51	5.26E-03
cellular component biogenesis (GO:0044085)	1.48	8.73E-06
import into cell (GO:0098657)	1.48	2.36E-02
aromatic compound biosynthetic process (GO:0019438)	1.48	3.54E-05
carboxylic acid transport (GO:0046942)	1.46	2.75E-02
organic acid transport (GO:0015849)	1.45	2.84E-02
nucleobase-containing small molecule metabolic process (GO:0055086)	1.45	1.95E-03
cellular component organization (GO:0016043)	1.43	2.84E-06
monoatomic cation transport (GO:0006812)	1.43	2.13E-02

cellular nitrogen compound metabolic process (GO:0034641)	1.42	1.47E-14
organic cyclic compound biosynthetic process (GO:1901362)	1.42	1.16E-04
monoatomic ion transport (GO:0006811)	1.41	1.06E-02
transmembrane transport (GO:0055085)	1.39	2.57E-07
small molecule biosynthetic process (GO:0044283)	1.39	2.41E-03
carbohydrate derivative metabolic process (GO:1901135)	1.38	3.97E-04
heterocycle biosynthetic process (GO:0018130)	1.38	1.12E-03
cellular component assembly (GO:0022607)	1.37	1.00E-02
organic substance transport (GO:0071702)	1.37	6.58E-05
amino acid metabolic process (GO:0006520)	1.36	2.10E-02
organonitrogen compound metabolic process (GO:1901564)	1.34	2.21E-09
organophosphate metabolic process (GO:0019637)	1.33	1.69E-02
nucleobase-containing compound metabolic process (GO:0006139)	1.32	5.46E-06
transport (GO:0006810)	1.32	8.75E-06
macromolecule metabolic process (GO:0043170)	1.32	1.35E-08
establishment of localization (GO:0051234)	1.31	1.22E-05
localization (GO:0051179)	1.31	1.87E-05
phosphate-containing compound metabolic process (GO:0006796)	1.28	1.35E-02
heterocycle metabolic process (GO:0046483)	1.26	4.51E-05
nitrogen compound metabolic process (GO:0006807)	1.26	4.18E-10

nucleic acid metabolic process (GO:0090304)	1.26	1.04E-02
cellular aromatic compound metabolic process (GO:0006725)	1.25	7.66E-05
phosphorus metabolic process (GO:0006793)	1.25	2.78E-02
primary metabolic process (GO:0044238)	1.25	5.25E-11
organic cyclic compound metabolic process (GO:1901360)	1.24	1.48E-04
cellular metabolic process (GO:0044237)	1.22	5.13E-11
cellular process (GO:0009987)	1.18	3.81E-21
organic substance metabolic process (GO:0071704)	1.16	4.21E-06
metabolic process (GO:0008152)	1.16	3.27E-07
biological_process (GO:0008150)	1.12	8.60E-16
regulation of RNA biosynthetic process (GO:2001141)	0.75	4.13E-02
regulation of DNA-templated transcription (GO:0006355)	0.75	4.12E-02
organic substance catabolic process (GO:1901575)	0.75	8.31E-03
catabolic process (GO:0009056)	0.74	4.04E-03
Unclassified (UNCLASSIFIED)	0.6	9.07E-16
cell communication (GO:0007154)	0.51	1.99E-03
signal transduction (GO:0007165)	0.45	2.78E-02
signaling (GO:0023052)	0.45	1.55E-02
intracellular signal transduction (GO:0035556)	0.37	1.77E-02
phosphorelay signal transduction system (GO:0000160)	0.33	1.01E-02
DNA transposition (GO:0006313)	0.31	2.92E-02
transposition (GO:0032196)	0.3	9.71E-03
benzene-containing compound metabolic process (GO:0042537)	0.12	2.71E-02
cellular response to xenobiotic stimulus (GO:0071466)	< 0.01	4.90E-03

xenobiotic metabolic process (GO:0006805)	< 0.01	4.87E-03
xenobiotic catabolic process (GO:0042178)	< 0.01	1.69E-02

Table S14 Transcripts less abundant in ∆*tolC tufA::kan* compared to ∆*tolC*

Gene ontology biological process	Fold Enrichment	p-value (FDR correction)
cytosine metabolic process (GO:0019858)	60.29	5.74E-02
NADH oxidation (GO:0006116)	60.29	5.36E-02
'de novo' UMP biosynthetic process (GO:0044205)	25.84	3.93E-02
pyrimidine ribonucleotide biosynthetic process (GO:0009220)	18.55	4.39E-02
pyrimidine nucleobase biosynthetic process (GO:0019856)	17.23	4.55E-02
aerobic electron transport chain (GO:0019646)	17.23	3.64E-02
pyrimidine ribonucleotide metabolic process (GO:0009218)	15.07	4.04E-02
pyrimidine nucleotide biosynthetic process (GO:0006221)	13.11	4.32E-02
siderophore-dependent iron import into cell (GO:0033214)	12.06	5.11E-02
nucleobase biosynthetic process (GO:0046112)	12.06	4.81E-02
pyrimidine nucleobase metabolic process (GO:0006206)	11.67	2.66E-02
pyrimidine nucleotide metabolic process (GO:0006220)	11.16	3.28E-02
iron import into cell (GO:0033212)	9.72	4.37E-02
nucleobase metabolic process (GO:0009112)	7.09	4.17E-02
iron ion transport (GO:0006826)	6.58	5.87E-02

pyrimidine-containing compound metabolic process (GO:0072527)	6.39	3.89E-02
monoatomic cation transport (GO:0006812)	3.7	4.31E-02

Table S15 Transcripts more abundant in ∆*tolC tufA::kan* compared to ∆*tolC*

Gene ontology biological process	Fold enrichment	p-value (FDR correction)
galactitol metabolic process (GO:0019402)	17.46	5.66E-05
behavior (GO:0007610)	17.46	9.16E-03
galactitol catabolic process (GO:0019404)	17.46	9.01E-03
response to mechanical stimulus (GO:0009612)	17.46	8.86E-03
multicellular organismal process (GO:0032501)	17.46	8.72E-03
mechanosensory behavior (GO:0007638)	17.46	8.59E-03
valine biosynthetic process (GO:0009099)	14.29	6.06E-08
valine metabolic process (GO:0006573)	14.29	5.66E-08
arginine catabolic process to succinate (GO:0019545)	13.97	2.97E-03
arginine catabolic process to glutamate (GO:0019544)	13.97	2.91E-03
alanine catabolic process (GO:0006524)	13.1	2.81E-02
methylgalactoside transport (GO:0015765)	13.1	2.77E-02
leucine biosynthetic process (GO:0009098)	13.1	7.82E-05
pyruvate family amino acid metabolic process (GO:0009078)	13.1	2.73E-02
glycine decarboxylation via glycine cleavage system (GO:0019464)	13.1	2.70E-02
L-alanine metabolic process (GO:0042851)	13.1	2.66E-02
leucine metabolic process (GO:0006551)	13.1	7.58E-05
branched-chain amino acid biosynthetic process (GO:0009082)	11.88	1.20E-13
branched-chain amino acid metabolic process (GO:0009081)	11.88	1.07E-13

isoleucine biosynthetic process (GO:0009097)	11.64	1.37E-07
isoleucine metabolic process (GO:0006549)	11.64	1.29E-07
galactose transmembrane transport (GO:0015757)	9.98	1.37E-02
glutamate metabolic process (GO:0006536)	8.22	1.36E-04
tricarboxylic acid cycle (GO:0006099)	8.15	4.15E-08
hexitol metabolic process (GO:0006059)	7.94	9.36E-03
alanine metabolic process (GO:0006522)	7.76	3.80E-02
non-proteinogenic amino acid catabolic process (GO:0170044)	7.28	1.40E-02
alditol catabolic process (GO:0019405)	6.79	2.16E-03
polyol catabolic process (GO:0046174)	6.11	4.15E-03
phenylacetate catabolic process (GO:0010124)	5.82	3.94E-02
dipeptide transmembrane transport (GO:0035442)	5.24	2.61E-02
alpha-amino acid biosynthetic process (GO:1901607)	5.2	8.90E-15
amino acid biosynthetic process (GO:0008652)	5.08	1.54E-16
non-proteinogenic amino acid metabolic process (GO:0170041)	4.76	3.74E-03
alditol metabolic process (GO:0019400)	4.76	3.67E-03
proteinogenic amino acid catabolic process (GO:0170040)	4.68	3.08E-05
L-amino acid catabolic process (GO:0170035)	4.68	2.97E-05
aerobic respiration (GO:0009060)	4.66	1.46E-05
alpha-amino acid catabolic process (GO:1901606)	4.37	1.58E-05
pyruvate metabolic process (GO:0006090)	4.37	1.38E-02
polyol metabolic process (GO:0019751)	4.25	8.83E-03
amino acid catabolic process (GO:0009063)	4.15	7.50E-06
alpha-amino acid metabolic process (GO:1901605)	4.1	3.19E-15

organic hydroxy compound catabolic process (GO:1901616)	4.03	1.78E-03
alcohol catabolic process (GO:0046164)	3.8	9.26E-03
amino acid metabolic process (GO:0006520)	3.64	1.89E-14
organic acid biosynthetic process (GO:0016053)	3.58	2.14E-11
carboxylic acid biosynthetic process (GO:0046394)	3.58	1.95E-11
proteinogenic amino acid biosynthetic process (GO:0170038)	3.41	4.48E-04
L-amino acid biosynthetic process (GO:0170034)	3.41	4.36E-04
proteinogenic amino acid metabolic process (GO:0170039)	3.33	6.00E-07
L-amino acid metabolic process (GO:0170033)	3.31	6.73E-07
aspartate family amino acid metabolic process (GO:0009066)	3.2	1.97E-02
carboxylic acid catabolic process (GO:0046395)	3.04	2.03E-07
dicarboxylic acid metabolic process (GO:0043648)	2.91	4.62E-03
carboxylic acid metabolic process (GO:0019752)	2.9	7.22E-18
organic acid catabolic process (GO:0016054)	2.88	7.03E-07
monocarboxylic acid catabolic process (GO:0072329)	2.79	7.50E-03
oxoacid metabolic process (GO:0043436)	2.77	4.01E-17
organic acid metabolic process (GO:0006082)	2.7	1.57E-16
cellular respiration (GO:0045333)	2.56	9.12E-03
small molecule biosynthetic process (GO:0044283)	2.54	3.18E-07
small molecule catabolic process (GO:0044282)	2.47	4.51E-07
energy derivation by oxidation of organic compounds (GO:0015980)	2.23	4.20E-02

monocarboxylic acid metabolic process (GO:0032787)	2.2	2.15E-03
organonitrogen compound catabolic process (GO:1901565)	2.17	2.46E-03
cellular catabolic process (GO:0044248)	2.09	9.43E-05
small molecule metabolic process (GO:0044281)	1.97	4.25E-11
organic substance catabolic process (GO:1901575)	1.86	2.05E-04
catabolic process (GO:0009056)	1.85	1.83E-04
organonitrogen compound metabolic process (GO:1901564)	1.44	9.85E-03
biological_process (GO:0008150)	1.14	1.95E-03
Unclassified (UNCLASSIFIED)	0.55	1.91E-03
heterocycle metabolic process (GO:0046483)	0.54	4.66E-03
nucleobase-containing compound metabolic process (GO:0006139)	0.47	1.91E-03
cellular nitrogen compound metabolic process (GO:0034641)	0.45	3.07E-05
macromolecule biosynthetic process (GO:0009059)	0.37	4.42E-04
macromolecule metabolic process (GO:0043170)	0.36	1.50E-07
nucleic acid metabolic process (GO:0090304)	0.31	4.38E-04
carbohydrate derivative biosynthetic process (GO:1901137)	0.2	1.13E-02
DNA metabolic process (GO:0006259)	0.13	1.94E-03
amide biosynthetic process (GO:0043604)	0.1	3.54E-02
translation (GO:0006412)	< 0.01	4.13E-02
ncRNA metabolic process (GO:0034660)	< 0.01	1.39E-02
peptide biosynthetic process (GO:0043043)	< 0.01	3.01E-02

Table S16 Transcripts that are more abundant in ∆*tolC tufA::kan* NMM compared to ∆*tolC tufA::kan*

Gene ontology biological process	Fold Enrichment	p-value (FDR correction)
heme oxidation (GO:0006788)	> 100	1.26E-04
tetrapyrrole catabolic process (GO:0033015)	> 100	2.03E-04
porphyrin-containing compound catabolic process (GO:0006787)	> 100	1.88E-04
aerobic electron transport chain (GO:0019646)	67.36	1.59E-03
nitrate assimilation (GO:0042128)	60.45	3.91E-06
nitrate metabolic process (GO:0042126)	60.45	3.42E-06
nitrogen cycle metabolic process (GO:0071941)	52.39	6.55E-06
reactive nitrogen species metabolic process (GO:2001057)	52.39	5.89E-06
formate oxidation (GO:0015944)	49.64	3.96E-03
pigment metabolic process (GO:0042440)	44.91	5.14E-03
heme metabolic process (GO:0042168)	44.91	4.88E-03
oxidative phosphorylation (GO:0006119)	42.87	5.37E-03
anaerobic electron transport chain (GO:0019645)	41	1.30E-06
porphyrin-containing compound metabolic process (GO:0006778)	39.29	6.71E-03
formate metabolic process (GO:0015942)	39.29	6.42E-03
respiratory electron transport chain (GO:0022904)	36.27	5.94E-10
tetrapyrrole metabolic process (GO:0033013)	32.52	1.10E-02
anaerobic respiration (GO:0009061)	28.15	7.29E-06
electron transport chain (GO:0022900)	23.98	1.40E-08
cellular respiration (GO:0045333)	23	1.36E-08
energy derivation by oxidation of organic compounds (GO:0015980)	20.07	3.57E-08
generation of precursor metabolites and energy (GO:0006091)	13.67	9.18E-07
oxoacid metabolic process (GO:0043436)	5.35	1.11E-03

organic acid metabolic process (GO:0006082)	5.21	1.30E-03
small molecule metabolic process (GO:0044281)	3.19	4.65E-02
